# Supplementary material for: Adipocyte lysoplasmalogenase TMEM86A regulates plasmalogen homeostasis and protein kinase A-dependent energy metabolism
Source: Nat Commun. 2022 Jul 14;13:4084. doi: 10.1038/s41467-022-31805-3 (PMC9283435; doi:10.1038/s41467-022-31805-3)
Supplement: Supplementary file 1 — Supplementary Information [file 41467_2022_31805_MOESM1_ESM.pdf]

Supplementary Information

Title: Adipocyte lysoplasmalogenase TMEM86A regulates plasmalogen homeostasis and protein kinase A-dependent energy metabolism

Yoon Keun Cho[1#], Young Cheol Yoon[1#], Hyeonyeong Im[1#], Yeonho Son[1], Minsoo Kim[1], Abhirup Saha[1], Cheoljun Choi[1], Jaewon Lee[1], Sumin Lee[1], Jae Hyun Kim[2], Yun Pyo Kang[1], Young-Suk Jung[3], Hong Koo Ha[4], Je Kyung Seong[5*], James G. Granneman[6*], Sung Won Kwon[1*], and Yun-Hee Lee[1*]

Supplementary Fig. 1-25

Supplementary Tables 1-2

Unprocessed gel blots for Supplementary Fig.

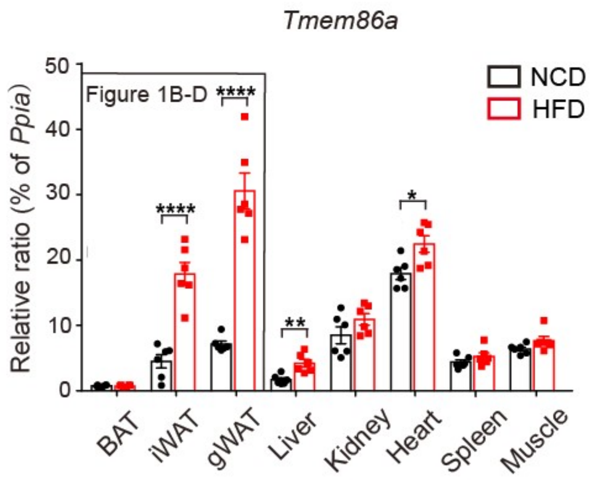

Supplementary Fig. 1. Effects of high-fat diet feeding on *Tmem86a* expression in various tissues. Related to Fig. 1

qPCR analysis of *Tmem86a* in BAT, iWAT, gWAT, liver, kidney, heart, spleen, and skeletal muscle (gastrocnemius muscle) of mice fed a normal chow diet (NCD) or high-fat diet (HFD) for 8 weeks. (iWAT: $p = 0.000063$; gWAT: $p = 0.000008$; Liver: $p = 0.003057$; Heart: $p = 0.015952$). $n = 6$. Each point represents a biological replicate. Data are presented as mean ± SEM. Statistical significance was determined using the unpaired, two-tailed t-test.

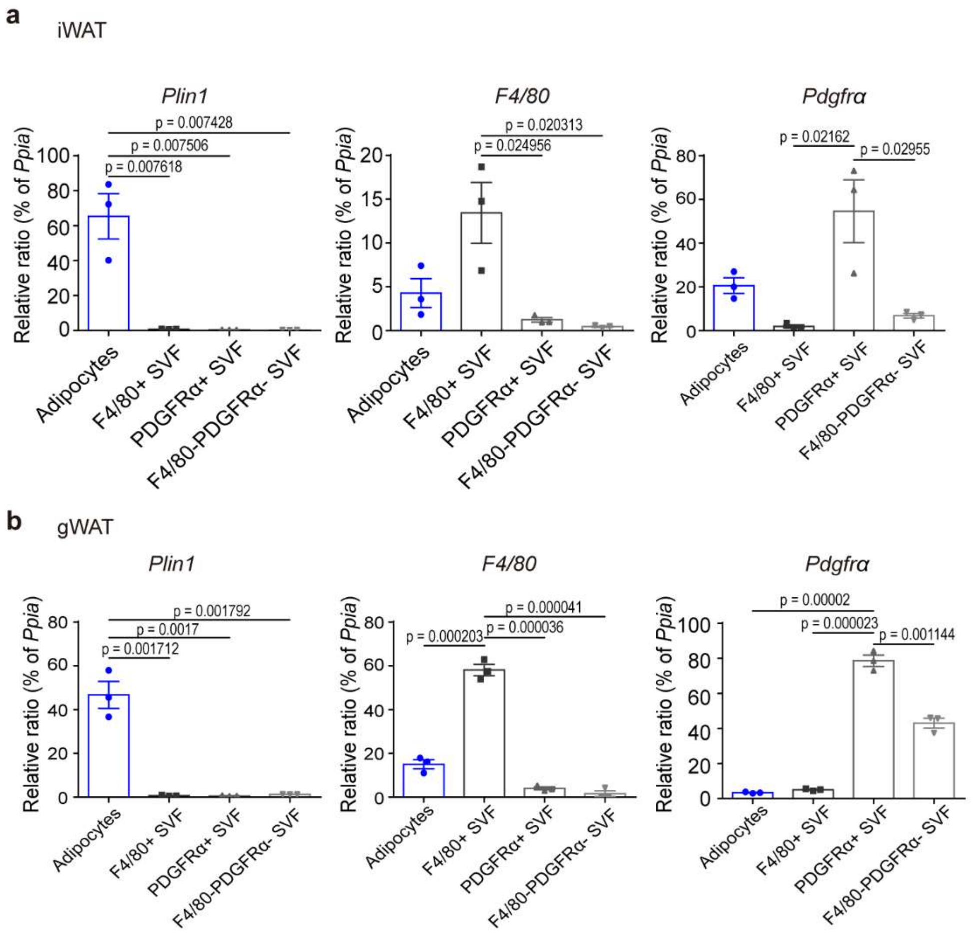

Supplementary Fig. 2. qPCR analyses of adipocytes and MACS-isolated cell fractions obtained from dissociated adipose tissue. Related to Fig. 1

a-b qPCR analyses of *Plin1* (an adipocyte marker*)*, *Pdgfra* (an adipocyte progenitor marker), and *Emr1* (a gene encoding F4/80, a macrophage marker) confirmed the isolation of each fraction in inguinal and gonadal white adipose tissue. n = 3. Each point represents a biological replicate. Data are presented as mean ± SEM. Statistical significance was determined using the unpaired, two-tailed t-test.

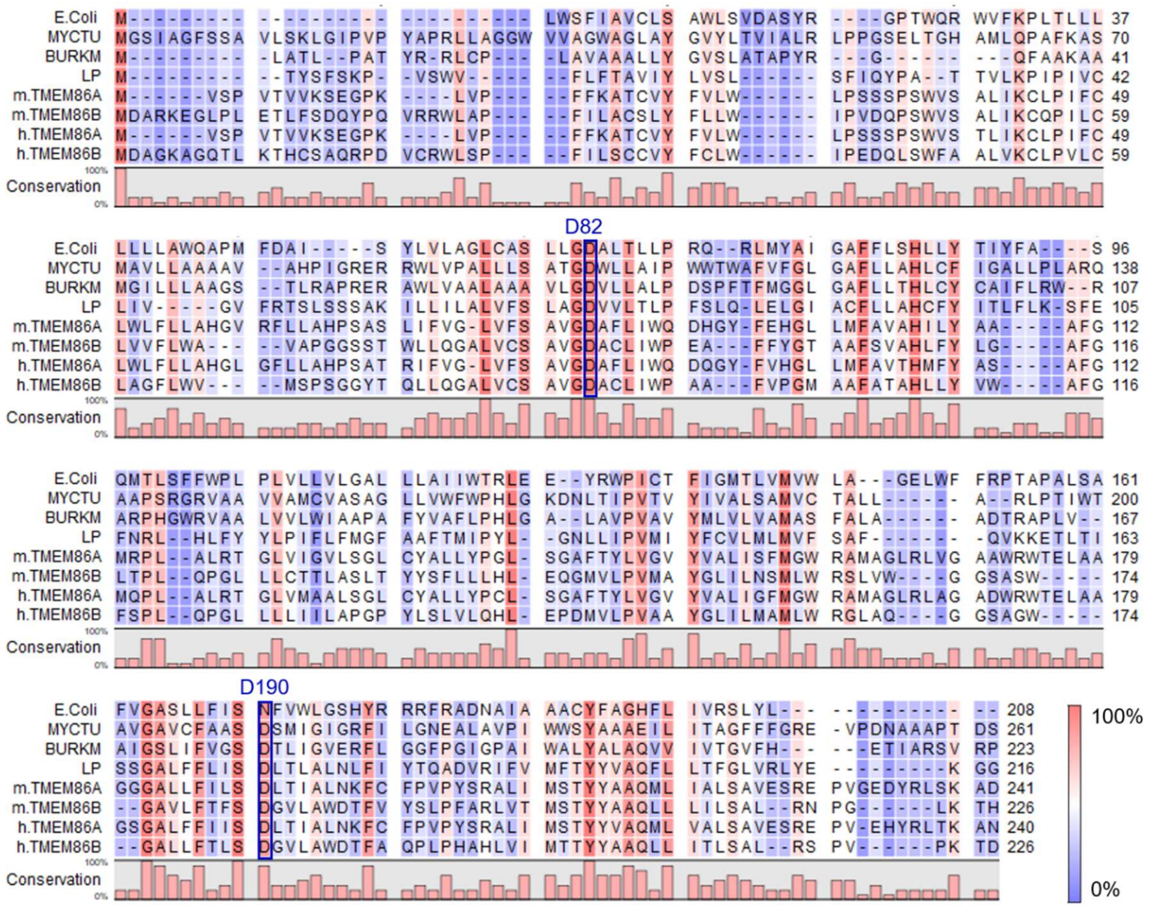

Supplementary Fig. 3. Sequence alignment of YhhN proteins. Related to Fig. 2

Multiple-sequence alignment of TMEM86A proteins from human, mouse, and bacterial species. The alignment was performed using CLC sequence viewer 8.0. Sequences are, from top to bottom, Escherichia coli (E.Coli), Mycobacterium tuberculosis (MYCTU), Burkolderia multivorans (BURKM), Legionella pneumophila (LP), *Mus musculus* (m.TMEM86A and m.TMEM86B) and *Homo sapiens* (h.TMEM86A and h.TMEM86B). Conserved sequences are highlighted according to the color bar indicated. Mutated sites of potential active sites (Asp 82 into Ala 82, D82A; Asp 190 into Ala 190, D190A) are indicated in blue box.

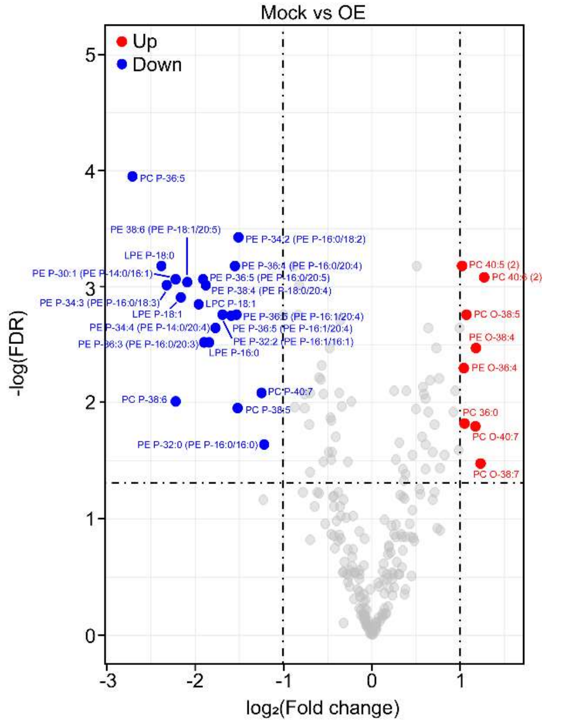

Supplementary Fig. 4. Volcano plot of untargeted phospholipid profiling in C3H10T1/2 adipocytes overexpressing TMEM86A or mock-transduced C3H10T1/2 adipocytes. Related to Fig. 2

Volcano plot of untargeted phospholipid profiling of C3H10T1/2 adipocytes overexpressing TMEM86A or mock-transduced C3H10T1/2 adipocytes. The exact values of fold change and FDR are provided in Supplementary Data 1. Blue points represent lipid species significantly reduced by TMEM86A overexpression while red points indicate lipids significantly increased by TMEM86A overexpression. ($FDR < 0.05$, $|\text{Fold change}| > 2$)

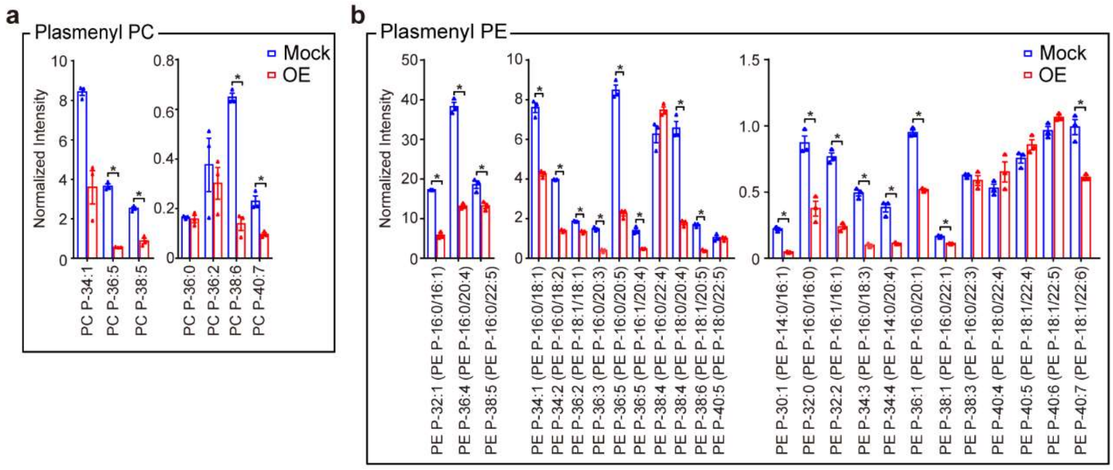

Supplementary Fig. 5. Normalized intensities of plasmalogen in C3H10T1/2 adipocytes overexpressing TMEM86A or mock-transduced C3H10T1/2 adipocytes. Related to Fig. 2

a-b Normalized intensities of plasmenyl PC (PC P-) and plasmenyl PE (PE P-) in C3H10T1/2 adipocytes overexpressing TMEM86A or mock-transduced C3H10T1/2 adipocytes (Intensity was normalized by median, *FDR < 0.05). n = 3. (**a** PC P-36:5: p < 0.000001; PC P-38:5: p = 0.002638; PC P-38:6: p = 0.002154; PC P-40:7: p = 0.001583; **b** PE P-32:1 (PE P-16:0/16:1): p = 0.000172; PE P-36:4 (PE P-16:0/20:4): p = 0.000013; PE P-38:5 (PE P-16:0/22:5): p = 0.008501; PE P-34:1 (PE P-16:0/18:1): p = 0.000158; PE P-34:2 (PE P-16:0/18:2): p = 0.000003; PE P-36:2 (PE P-18:1/18:1): p = 0.000447; PE P-36:3 (PE P-16:0/20:3): p = 0.000375; PE P-36:5 (PE P-16:0/20:5): p = 0.000036; PE P-36:5 (PE P-16:1/20:4): p = 0.000152; PE P-38:4 (PE P-18:0/20:4): p = 0.000060; PE P-38:6 (PE P-18:1/20:5): p = 0.000044; PE P-30:1 (PE P-14:0/16:1): p = 0.000038; PE P-32:0 (PE P-16:0/16:0): p = 0.006707; PE P-32:2 (PE P-16:1/16:1): p = 0.000158; PE P-34:3 (PE P-16:0/18:3): p = 0.000057; PE P-34:4 (PE P-14:0/20:4): p = 0.000260; PE P-36:1 (PE P-16:0/20:1): p = 0.000018; PE P-38:1 (PE P-16:0/22:1): p = 0.000673; PE P-40:7 (PE P-18:1/22:6): p = 0.001582) Each point represents a biological replicate. Data are presented as mean ± SEM. Statistical significance was determined using the unpaired, two-tailed t-test.

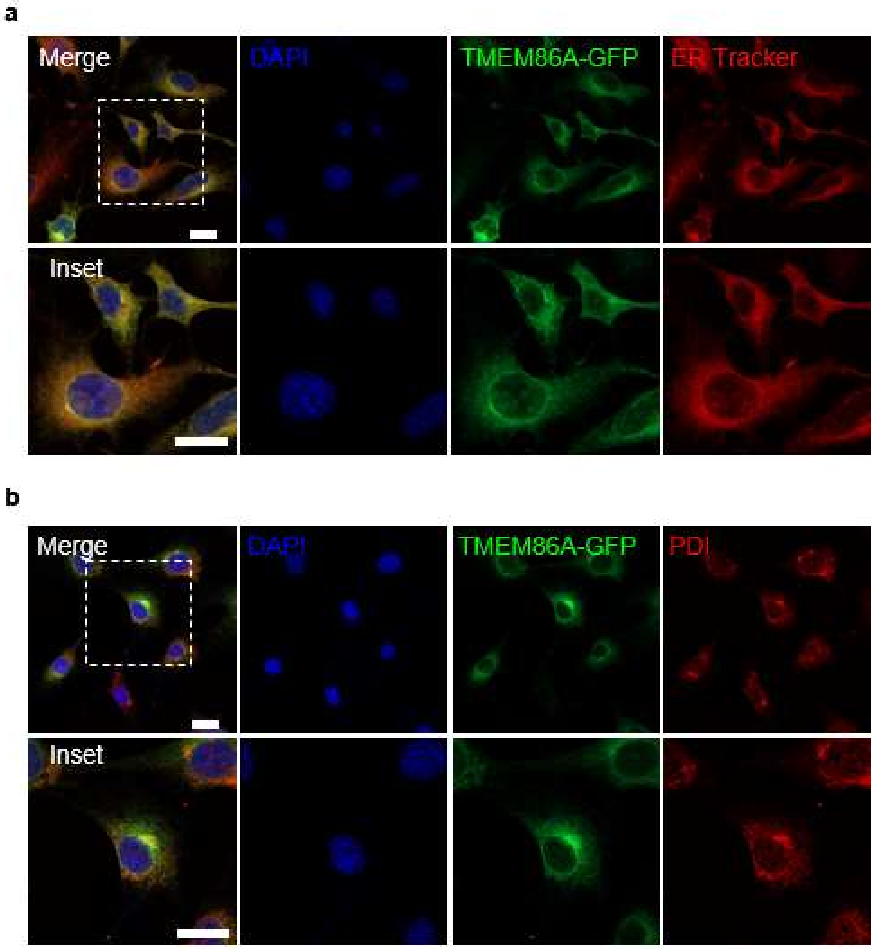

Supplementary Fig. 6. TMEM86A was mainly localized in Endoplasmic Reticulum (ER). Related to Fig. 2

a-b Immunofluorescence imaging of C3H10T1/2 cells overexpressing GFP-tagged TMEM86A. Cells were stained with ER-Tracker (a) or protein disulfide-isomerase (PDI, b). Scale bars = 10 μm. n = 3.

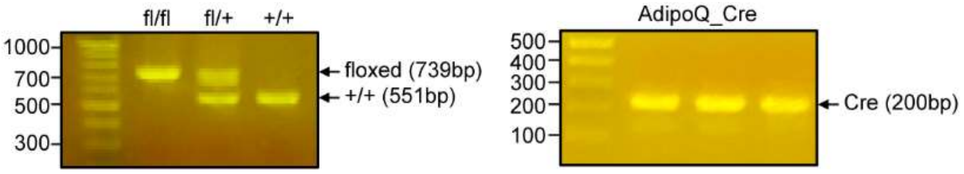

Supplementary Fig. 7. Genotyping results of *Tmem86a* floxed and AdipoqCre mice. Related to Fig. 3

Genotyping results of *Tmem86a* floxed and AdipoqCre mice. *Tmem86a* floxed mice was generated by breeding *Tmem86a*$^{tm1a(KOMP)Mbp}$ mice with ACTB-FLPe (B6.Cg-Tg(ACTFLPe)9205Dym/J) mice. Genotype of *Tmem86a* floxed mice was confirmed by using two primers (F': CTTACTCCAGTTCCACACAGC and R': CCAGGCTTCAGGTCTAAACATC). The two primers amplified a 739 bp mutant amplicon (floxed) and a 551 bp wild-type amplicon (+/+) from mice carrying a deletion in a gene of interest. Genotyping was performed for all mice used in KO mouse study.

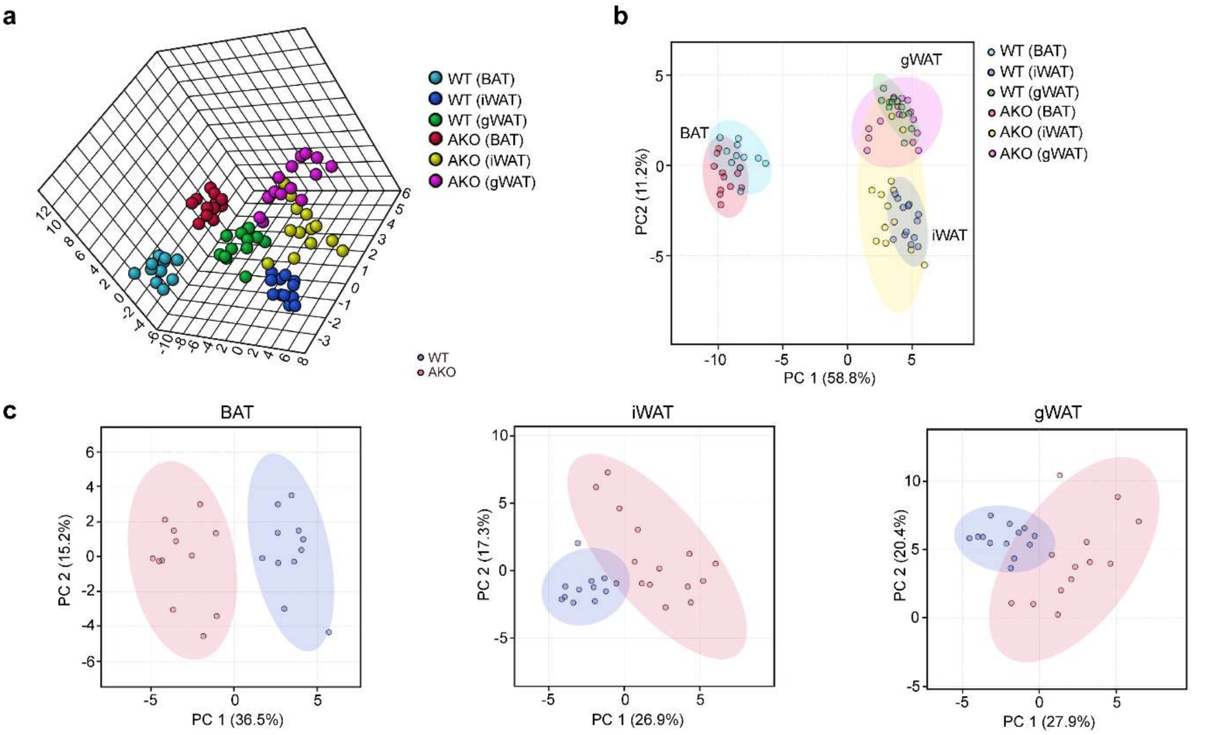

Supplementary Fig. 8. PCA plots: untargeted phospholipid profiling of adipose tissue from WT and TMEM86A AKO mice. Related to Fig. 3

a-b 3D and 2D PCA plots showing a separation of clusters of BAT, iWAT and gWAT of WT and TMEM86A AKO mice. **c** PCA plots indicating separation of WT and TMEM86A AKO samples.

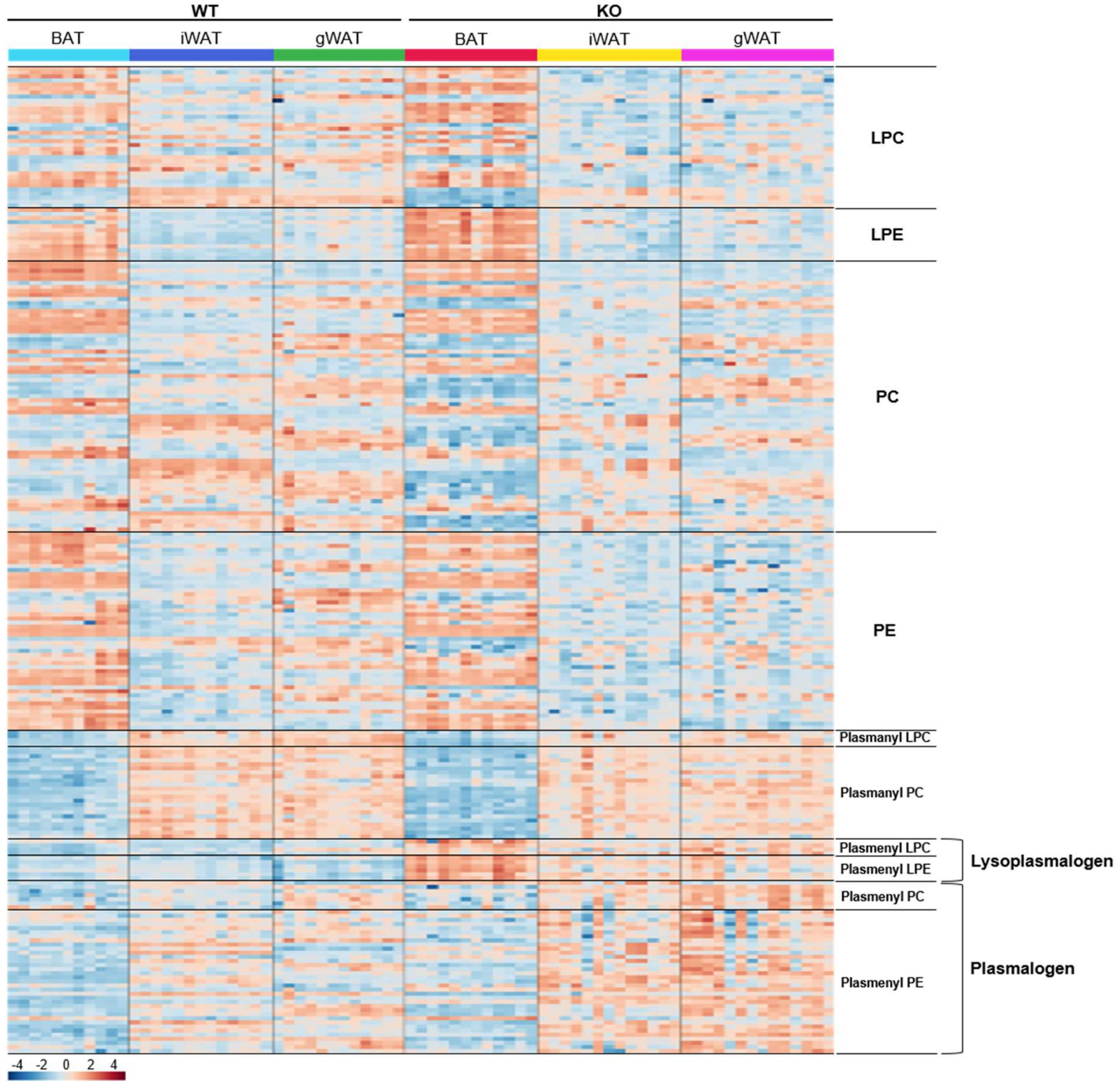

Supplementary Fig. 9. Heatmap analysis of untargeted phospholipid profiling in adipose tissue of WT and TMEM86A AKO mice. Related to Fig. 3

Entire heatmap of untargeted phospholipid profiling from BAT, iWAT and gWAT of WT and TMEM86A AKO mice. BAT WT: n = 11, KO: n = 12; iWAT WT: n = 13, KO: n = 13; gWAT WT: n = 12, KO: n = 14.

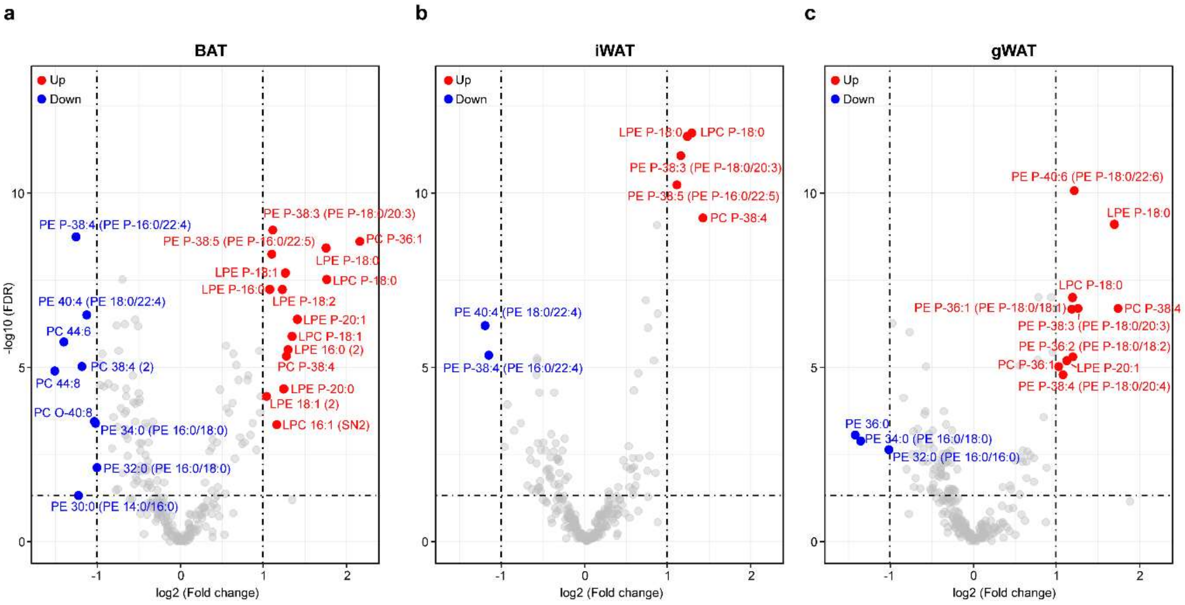

Supplementary Fig. 10. Volcano plots of untargeted phospholipid profiling from adipose tissue of WT and TMEM86A AKO mice. Related to Fig. 3

a-c Volcano plots of WT and TMEM86A AKO revealed that significantly increased phospholipids were lysoplasmalogen and plasmalogen (│Fold change│ > 2).

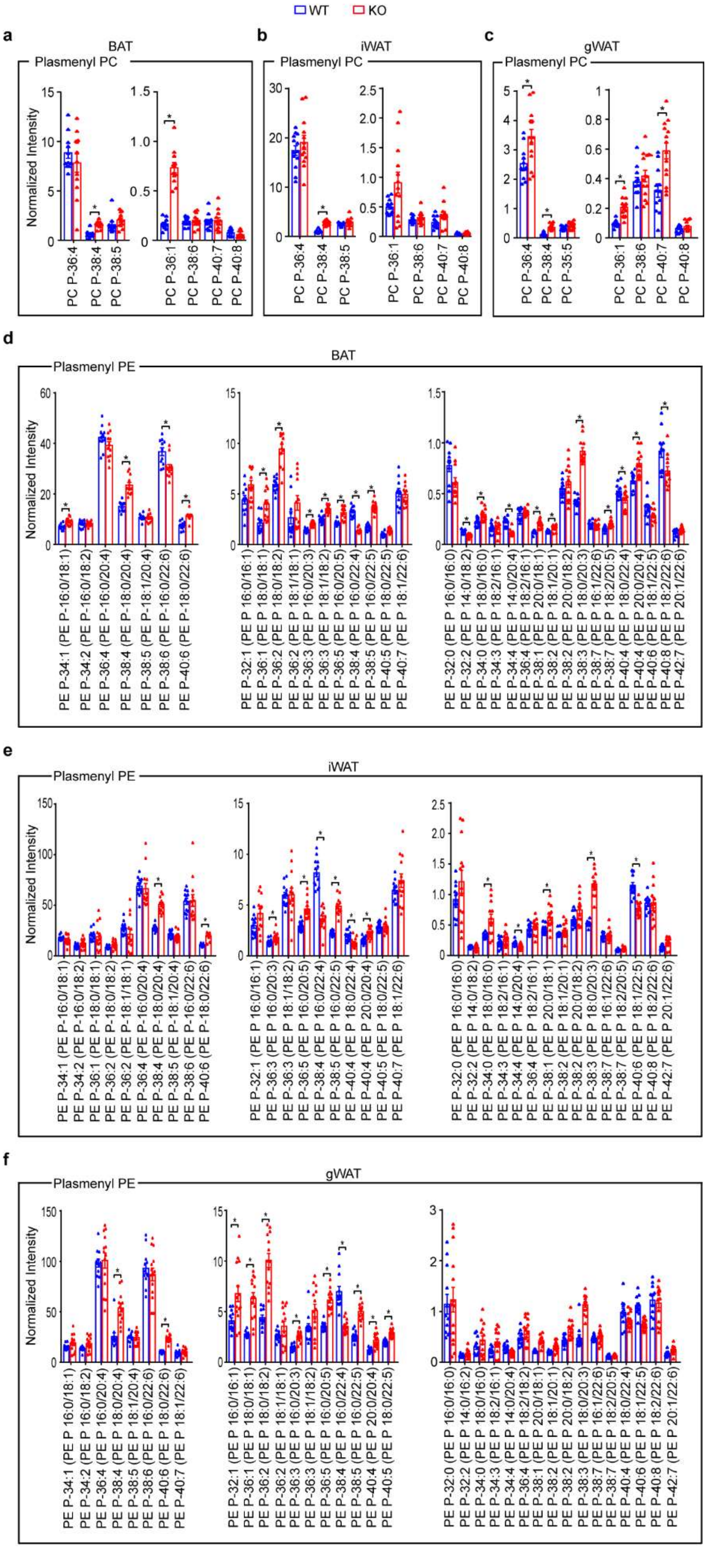

Supplementary Fig. 11. Normalized intensities of plasmalogen in adipose tissue of WT and TMEM86A AKO. Related to Fig. 3

a-f Normalized intensities of plasmenyl PC (PC P-) and plasmenyl PE (PE P-) in BAT, iWAT, and gWAT of WT and TMEM86A AKO mice (intensity was normalized by median, *FDR < 0.05). BAT WT: n = 11, KO: n = 12; iWAT WT: n = 13, KO: n = 13; gWAT WT: n = 12, KO: n = 14. (**a** PC P-38:4: p = 0.000006; PC P-36:1: p < 0.000001; **b** PC P-38:4: p < 0.000001; **c** PC P-36:4: p = 0.008831; PC P-38:4: p < 0.000001; PC P-36:1: p < 0.000001; PC P-40:7: p = 0.002159; **d** PE P-34:1 (PE P-16:0/18:1): p = 0.000684**;** PE P-38:6 (PE P-16:0/22:6): p = 0.003684; PE P-40:6 (PE P-18:0/22:6): p = 0.000046; PE P-36:1 (PE P-18:0/18:1): p = 0.000795; PE P-36:2 (PE P-18:0/18:2): p = 0.000001**;** PE P-36:3 (PE P-18:1/18:2): p = 0.000007; PE P-36:5 (PE P-16:0/20:5): p = 0.000033; PE P-32:2 (PE P-14:0/18:2): p = 0.00002; PE P-34:0 (PE P-18:0/16:0): p = 0.002685; PE P-38:1 (PE P-20:0/18:1): p = 0.000003; PE P-38:2 (PE P-18:1/20:1): p = 0.002734; PE P-38:7 (PE P-18:2/20:5): p = 0.000351; PE P-40:4 (PE P-18:0/22:4): p = 0.015587; PE P-40:4 (PE P-20:0/20:4): p = 0.000918; PE P-40:8 (PE P-18:2/22:6): p = 0.013469; p < 0.000001 for PE P-38:4 (PE P-18:0/20:4), PE P-36:3 (PE P-16:0/20:3), PE P-38:4 (PE P-16:0/22:4), PE P-38:5 (PE P-16:0/22:5), PE P-34:4 (PE P-14:0/20:4), and PE P-38:3 (PE P-18:0/20:3); **e** PE P-36:3 (PE P-16:0/20:3): p = 0.008062; PE P-40:4 (PE P-18:0/22:4): p = 0.000344; PE P-40:4 (PE P-20:0/20:4): p = 0.000221; PE P-34:0 (PE P-18:0/16:0): p = 0.002519; PE P-34:4 (PE P-14:0/20:4): p = 0.003378; PE P-38:1 (PE P-20:0/18:1): p = 0.001794**;** p < 0.000001 for PE P-38:4 (PE P-18:0/20:4), PE P-40:6 (PE P-18:0/22:6), PE P-36:5 (PE P-16:0/20:5), PE P-38:4 (PE P-16:0/22:4), PE P-38:5 (PE P-16:0/22:5), PE P-38:3 (PE P-18:0/20:3), and PE P-40:6 (PE P-18:1/22:5); **f** PE P-38:4 (PE P-18:0/20:4): p = 0.000001; PE P-32:1 (PE P-16:0/16:1): p = 0.006800; PE P-40:4 (PE P-20:0/20:4): p = 0.000027; p < 0.000001 for PE P-40:6 (PE P-18:0/22:6), PE P-36:1 (PE P-18:0/18:1), PE P-36:2 (PE P-18:0/18:2), PE P-36:3 (PE P-16:0/20:3), PE P-36:5 (PE P-16:0/20:5), PE P-38:4 (PE P-16:0/22:4), PE P-38:5 (PE P-16:0/22:5), and PE P-40:5 (PE P 18:0/22:5)). Each point represents a biological replicate. Data are presented as mean ± SEM. Statistical significance was determined using the unpaired, two-tailed t-test.

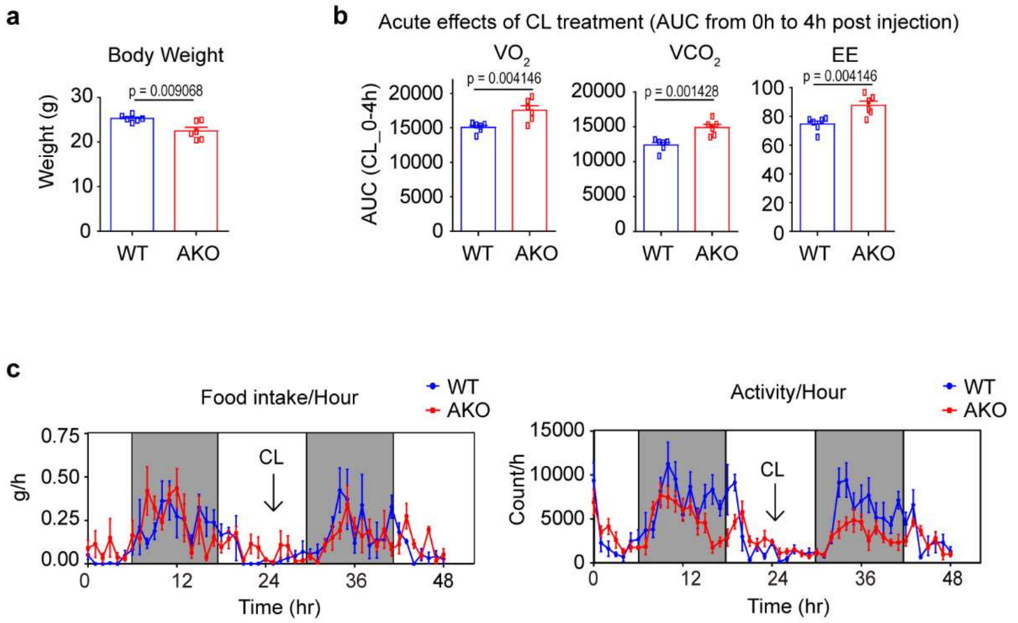

Supplementary Fig. 12. Adipocyte-specific knockout of TMEM86A enhances energy expenditure. Related to Fig. 4

a Body Weight of WT and TMEM86A AKO mice. n = 6. **b** Area under the curve (AUC) values of VO_2, VCO_2, and energy expenditure (EE) of initial 4 hours of CL treatment (from 0 to 4 hours post injection). n = 6. **c** Analysis of food intake and activity. n = 6. Each point represents a biological replicate. Data are presented as mean ± SEM. Statistical significance was determined using the unpaired, two-tailed t-test in **a** and **b.**

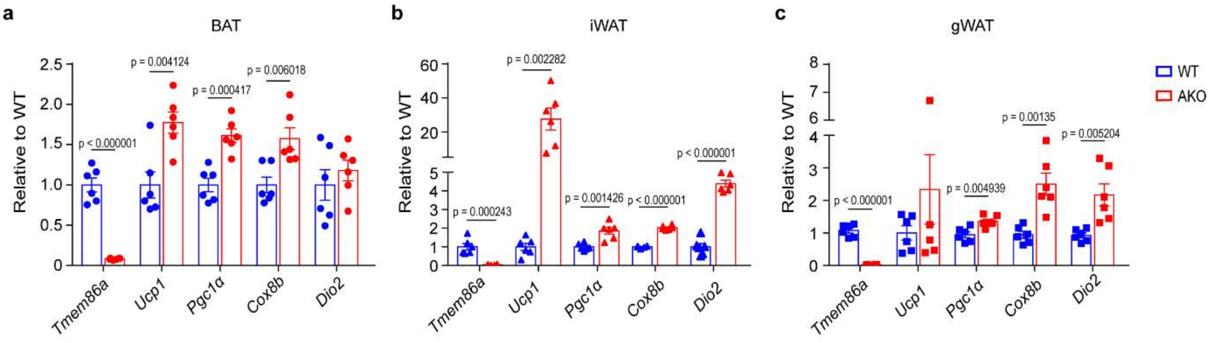

Supplementary Fig. 13. TMEM86A enhanced browning markers in adipose tissue. Related to Fig. 4

a-c qPCR analyses of BAT, iWAT, and gWAT from WT and TMEM86A AKO mice. n = 6. Each point represents a biological replicate. Data are presented as mean ± SEM. Statistical significance was determined using the unpaired, two-tailed t-test in **a-c.**

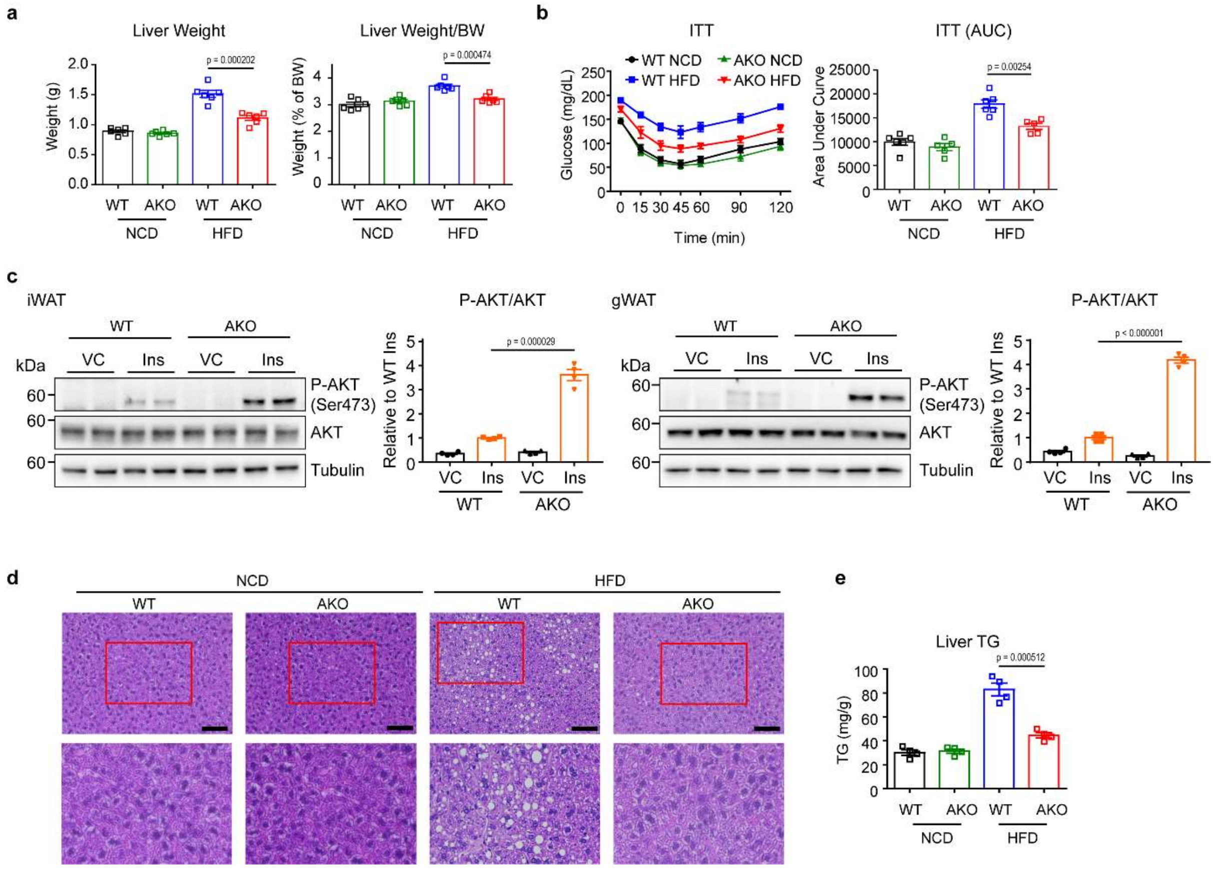

Supplementary Fig. 14. Adipocyte-specific TMEM86A KO protects mice against HFD-induced metabolic dysfunction. Related to Fig. 6

a Liver weight of WT or TMEM86A AKO fed a NCD or HFD for 8 weeks. n = 6 **b** Intraperitoneal insulin tolerance test of WT or TMEM86A AKO mice fed a NCD or HFD for 8 weeks. n = 6 for WT, n = 5 for TMEM86A AKO. **c** Immunoblot analysis of p-AKT and AKT in iWAT, and gWAT of WT or TMEM86A AKO mice fed a NCD or HFD for 8 weeks, acutely treated with insulin (Ins, 0.75 units/kg body weight) or vehicle controls (VC) via inferior vena cava. n = 4. **d** Representative images of H&E stained paraffin sections of liver from WT and TMEM86A AKO mice fed a NCD or HFD for 8 weeks. Scale bars = 50 μm. n = 3. **e** Triglyceride levels in liver from WT and TMEM86A AKO mice fed a NCD or HFD for 8 weeks. n = 4. Each point represents a biological replicate. Data are presented as mean ± SEM. Statistical significance was determined using the unpaired, two-tailed t-test in **a-c.**

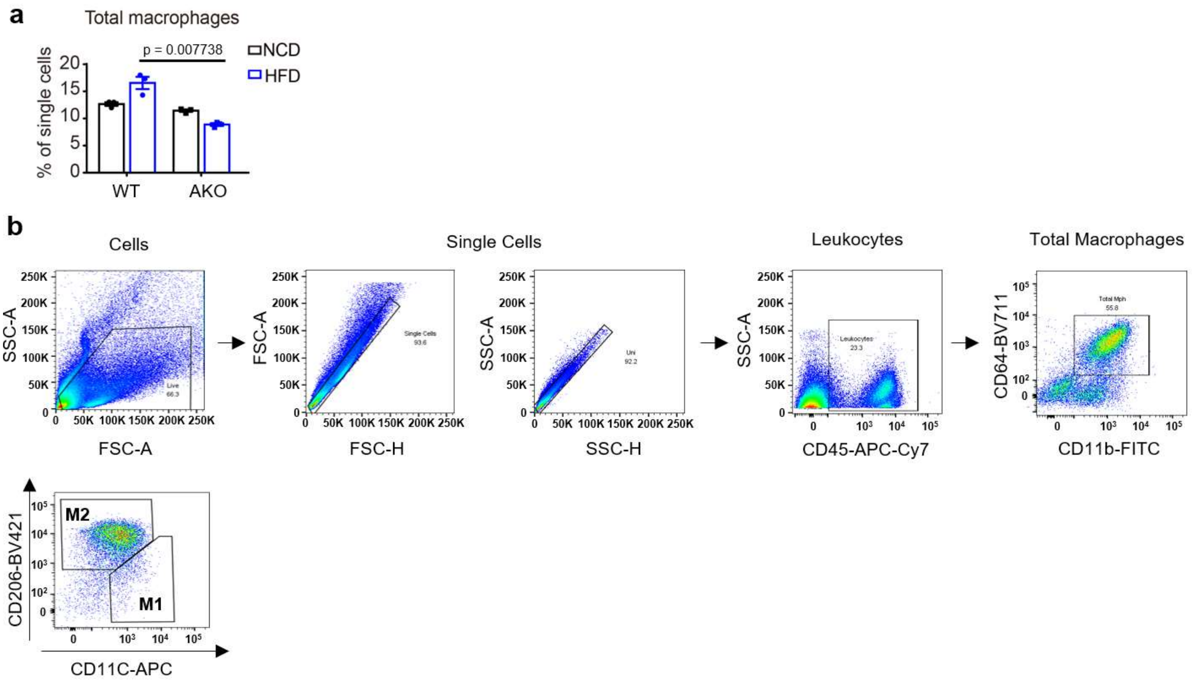

Supplementary Fig. 15. Deletion of TMEM86A in adipocyte prevents the accumulation of total macrophages caused by high-fat diet. Related to Fig. 6

a Flow cytometric analysis of CD45+CD11b+CD64+ cells (Total macrophages) from gWAT of WT or TMEM86A AKO mice. n = 3. **b** Flow cytometry analysis was conducted with SVF obtained from gWAT of WT or TMEM86A AKO mice. Gating strategy for analysis of SVF was suggested as following. Each point represents a biological replicate. Data are presented as mean ± SEM. Statistical significance was determined using the unpaired, two-tailed t-test in **a.**

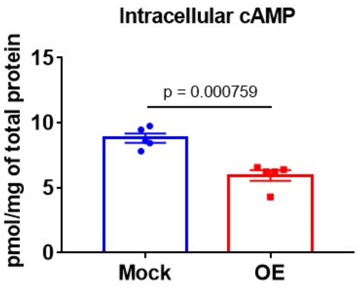

Supplementary Fig. 16. Effects of TMEM86A overexpression on intracellular cAMP levels in C3H10T1/2 adipocytes. Related to Fig. 7

Intracellular cAMP levels in TMEM86A overexpressing C3H10T1/2 adipocytes or controls. n = 5. Each point represents a biological replicate. Data are presented as mean ± SEM. Statistical significance was determined using the unpaired, two-tailed t-test.

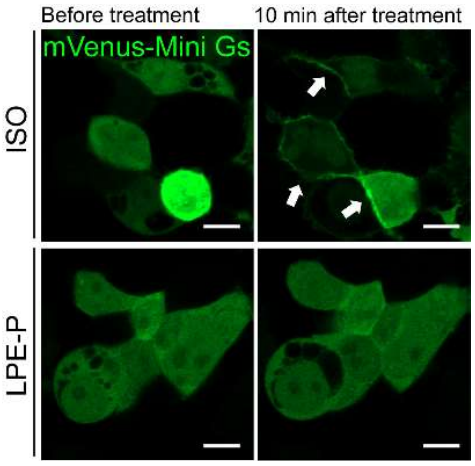

Supplementary Fig. 17. Representative live cell imaging of HEK293T overexpressing mVenus-tagged mini Gs and beta-1-adrenergic receptor. Related to Fig. 7

Representative live cell imaging of HEK293T cells overexpressing mVenus-tagged mini Gs proteins and beta-1-adrenergic receptor treated with either isoproterenol (ISO, 10 μM) or LPE P-18:0 (LPE-P, 10 μM). n = 3. Scale bars = 10 μm.

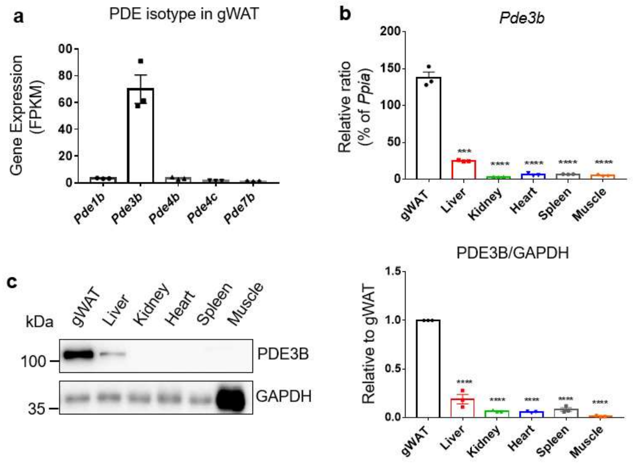

Supplementary Fig. 18. Analysis of PDE3B expression levels. Related to Fig. 7

a PDE isotype expression levels obtained from RNA-seq analysis of gonadal white adipose tissue (gWAT) of WT mice. n = 3. **b** qPCR analysis of *Pde3b* in gWAT, liver, kidney, heart, spleen, and skeletal muscle (gastrocnemius muscle) of WT mice. n = 3. (Liver: p = 0.000124; Kidney: p = 0.000061; Heart: p = 0.000069; Spleen: p = 0.000068; Muscle: p = 0.000066) **c** Immunoblot analysis of PDE3B in gWAT, liver, kidney, heart, spleen, and skeletal muscle (gastrocnemius muscle) of WT mice. n = 3. (Liver: p = 0.000078; Kidney: p < 0.000001; Heart: p < 0.000001; Spleen: p < 0.000001; Muscle: p < 0.000001). Each point represents a biological replicate. Data are presented as mean ± SEM. Statistical significance was determined using the unpaired, two-tailed t-test.

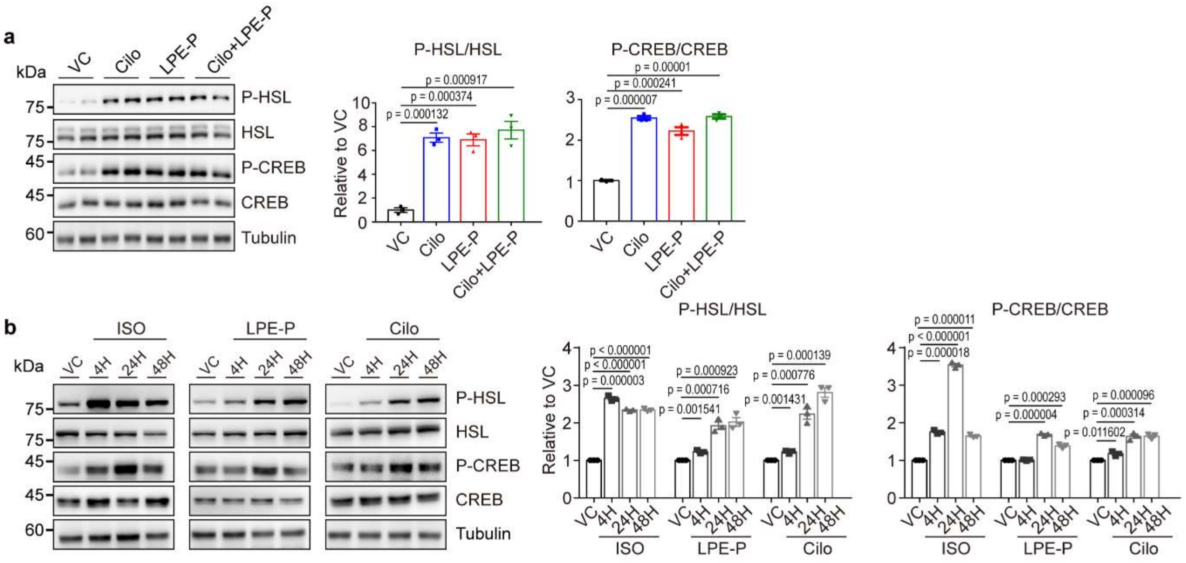

Supplementary Fig. 19. Effects of LPE P-18:0 and PDE3B inhibition on PKA signaling pathway in adipocytes. Related to Fig. 7

a Immunoblot analysis of C3H10T1/2 adipocytes treated with vehicle control (VC), cilostamide (cilo, 10 μM), LPE P-18:0 (LPE-P, 10 μM), or cilo (10 μM) + LPE-P (10 μM) for 24 h. n= 3. **b** Immunoblot analysis of C3H10T1/2 adipocytes treated with isoproterenol (ISO, 10 μM), LPE-P (10 μM), or cilo (10 μM) for 4, 24, or 48 h. n = 3. Each point represents a biological replicate. Data are presented as mean ± SEM. Statistical significance was determined using the unpaired, two-tailed t-test in **a** and **b.**

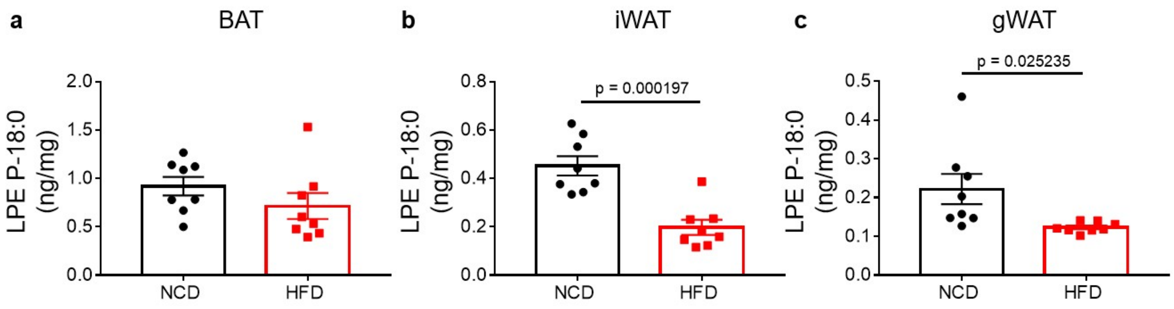

Supplementary Fig. 20. Quantification of LPE P-18:0 in BAT, iWAT, and gWAT of mice fed a NCD or HFD for 8 weeks

a-c Targeted lipidomics analysis of LPE P-18:0 in BAT, iWAT, and gWAT of mice fed a NCD or HFD for 8 weeks. n = 8. Each point represents a biological replicate. Data are presented as mean ± SEM. Statistical significance was determined using the unpaired, two-tailed t-test in **a-c.**

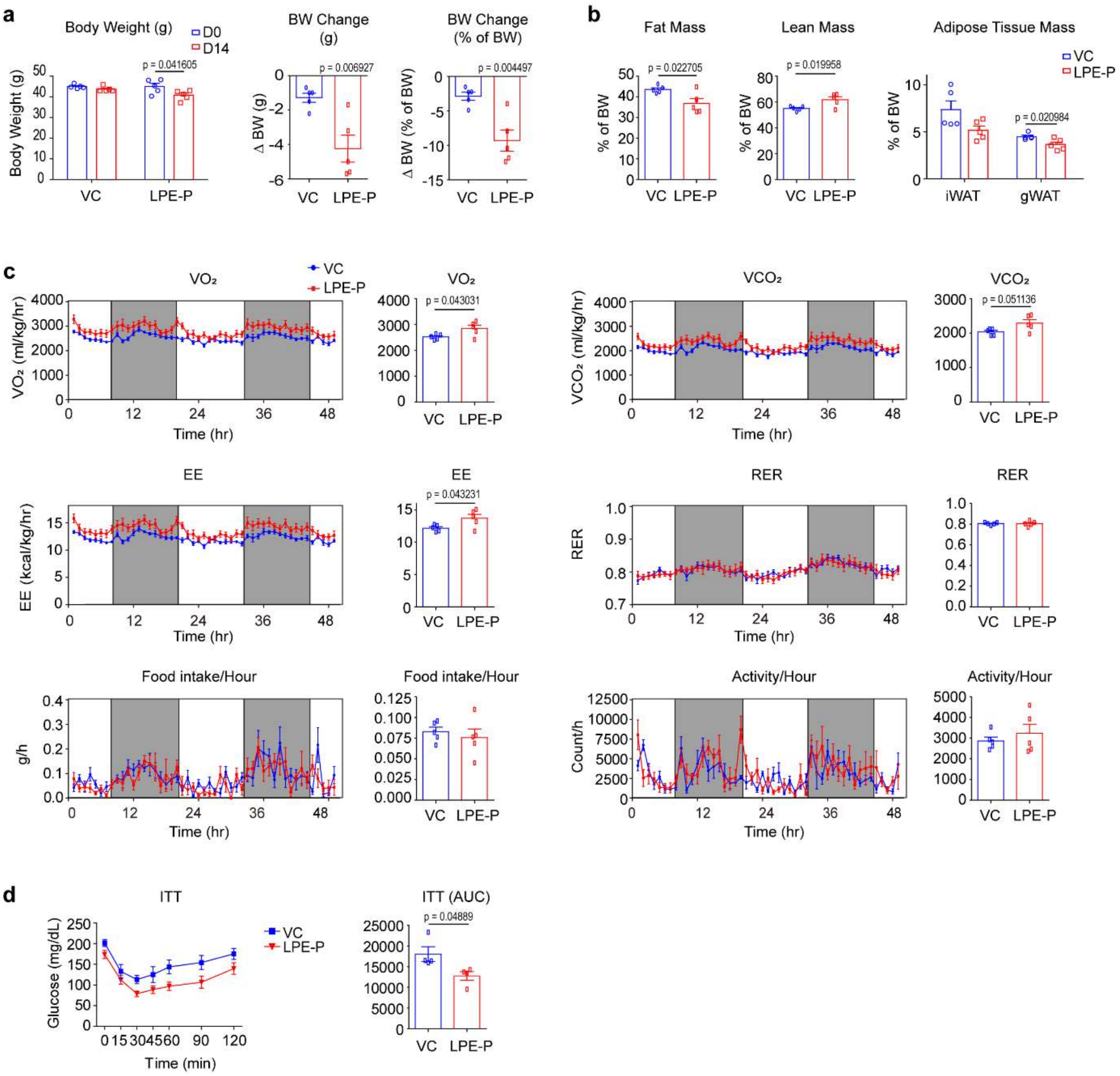

Supplementary Fig. 21 LPE P-18:0 treatment increases energy expenditure and protects against diet-induced obesity. Related to Fig. 8

a-b Body weight analysis, body composition, and adipose tissue mass of WT mice fed a HFD for 8 weeks treated with either vehicle controls (VC) or LPE P-18:0 (LPE-P, 200 *μ*g/kg/day) for 14 days. n = 6. **c** Indirect calorimetry analysis of WT mice fed a HFD for 8 weeks treated with either VC or LPE P-18:0 (200 *μ*g/kg/day) for 14 days **d** Intraperitoneal insulin tolerance test of mice fed a HFD for 8 weeks, treated with VC or LPE P-18:0. n = 4. Each point represents a biological replicate. Data are presented as mean ± SEM. Statistical significance was determined using the unpaired, two-tailed t-test in **a-d.**

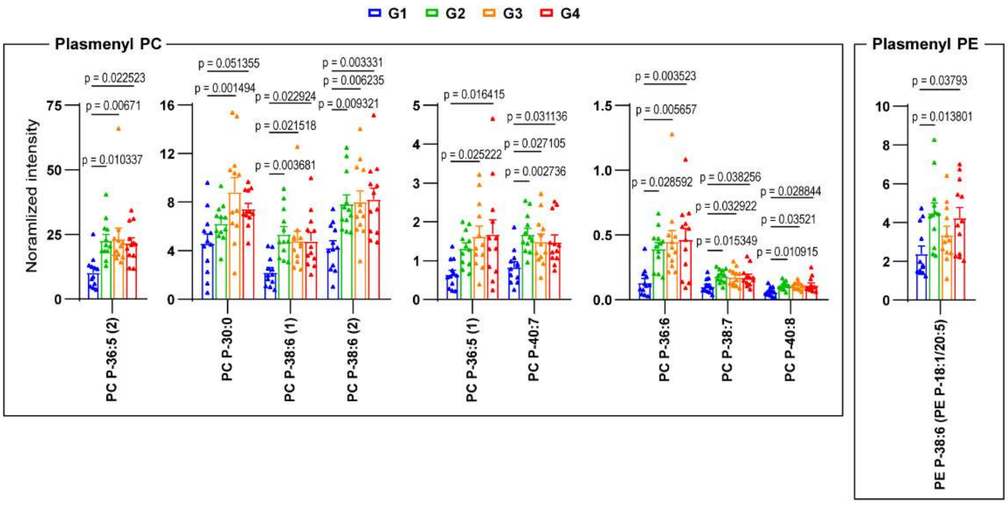

Supplementary Fig. 22. Normalized intensities of plasmalogen in human subcutaneous adipose tissue. Related to Fig. 8

Normalized intensities of plasmalogen in human subcutaneous adipose tissue (intensity was normalized by median, *FDR < 0.05). n= 48. Each point represents a biological replicate. Data are presented as mean ± SEM. Statistical significance was determined using ANOVA (one-way, Tukey's HSD post-hoc analysis).

a

Parameter	Value (Untargeted lipidomics)	Value (Targeted-SIM)
**ESI Polarity**	Positive mode	Positive mode
**SHEATH GAS PRESSURE**	60 arbitrary units	60 arbitrary units
**AUX GAS FLOW**	25 arbitrary units	25 arbitrary units
**SWEEP GAS FLOW**	2 arbitrary units	2 arbitrary units
**SPRAY VOLTAGE**	3.6 kV	3.6 kV
**Capillary temperature**	300 °C	300 °C
**S-lens RF LEVEL**	50	50
**AUS GAS HEATER TEMPERATURE**	370 °C	370 °C
**NUMBER OF SCANS**	1 spectra/s	1 spectra/s
**MS1 MASS RANGE**	*m/z* 400-1000	*m/z* 466.32920 (LPE P-18:0; $C_{23}H_{48}NO_6P$) with isolation window 0.4 *m/z*
**MICROSCANS (MS1)**	1	1
**RESOLUTION (MS1)**	70,000 FWHM (*m/z* 200)	140,000 FWHM (*m/z* 200)
**AGC TARGET (MS1)**	1e6	1e6
**MAXIMUM IT (MS1)**	100 ms	100 ms
**SPECTRUM DATA TYPE (MS1)**	Profile	Profile
**MICROSCANS (MS2)**	1	NA
**RESOLUTION (MS2)**	17,500 FWHM (*m/z* 200)	NA
**AGC TARGET (MS2)**	1e5	NA
**MAXIMUM IT (MS2)**	50 ms	NA
**LOOP COUNT (MS2)**	4	NA
**MSX COUNT (MS2)**	1	NA
**Top N (MS2)**	4	NA
**ISOLATION WINDOW (MS2)**	1.0 *m/z*	NA
**ISOLATION OFFSET (MS2)**	0.0 *m/z*	NA
**NORMALIZED COLLISION ENERGY (MS2)**	20, 30	NA
**SPECTRUM DATA TYPE (MS2)**	Profile	NA

b

Parameter		Value
**ESI Polarity**		Positive mode
**Gas Temp**		325°C
**Gas Flow**		8 L/min
**Nebulizer**		25 psi
**SheathGasHeater**		350
**SheathGasFlow**		11
**Capillary**		3500 V
**VCharging**		1000
**MRM transition 1 (LPE P-18:0)**	**Precursor Ion**	466.33 *m/z*
	**Product Ion**	294.31 *m/z*
	**Dwell time**	1000 ms
	**Fragmentor**	150 V
	**Collision energy**	20 V
**MRM transition 2 (LPE 17:1) (Internal standard)**	**Precursor Ion**	466.29 *m/z*
	**Product Ion**	325.27 *m/z*
	**Dwell time**	100 ms
	**Fragmentor**	120 V
	**Collision energy**	12 V

Supplementary Fig. 23. Mass spectrometry condition

a Instrumental parameters of untargeted lipidomics and LPE P-18:0 targeted analysis (Targeted-SIM mode) **b** Instrumental parameters of LPE P-18:0 quantitative analysis in mouse serum (MRM mode)

LPC O-18:1 (Retention time 1.993 min; *m/z* 508.37534; Adduct form [M+H]$^+$)

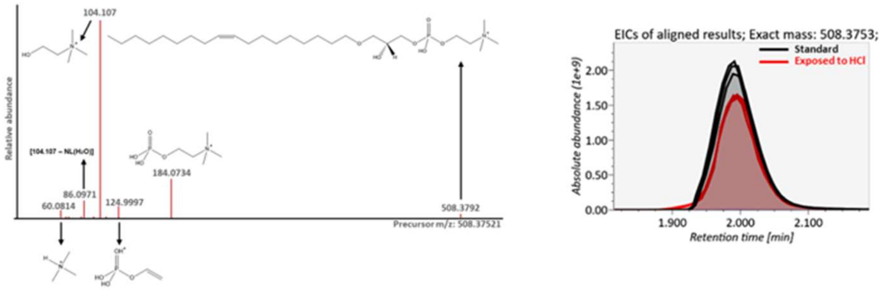

PC O-34:1 (PC O-16:0/18:1) (Retention time 6.344 min; *m/z* 746.60522; Adduct form [M+H]$^+$)

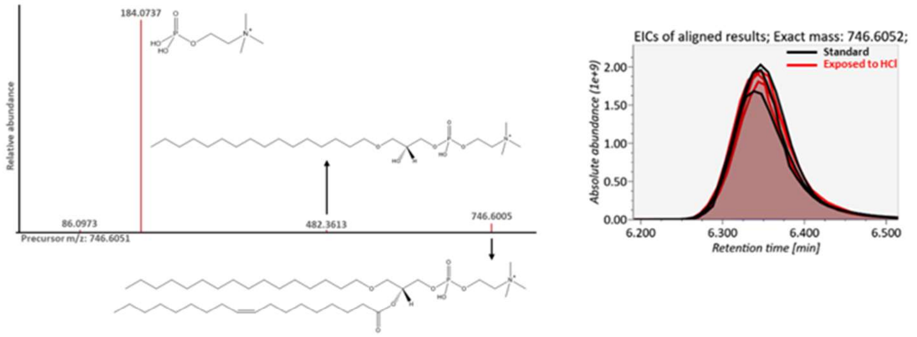

LPE O-16:0 (Retention time 1.882 min; *m/z* 440.31491; Adduct form [M+H]$^+$)
Because there was not commercially available plasmanyl LPE standard, LPE O-16:0 was generated from PE O-34:1 standard by phospholipase A2.

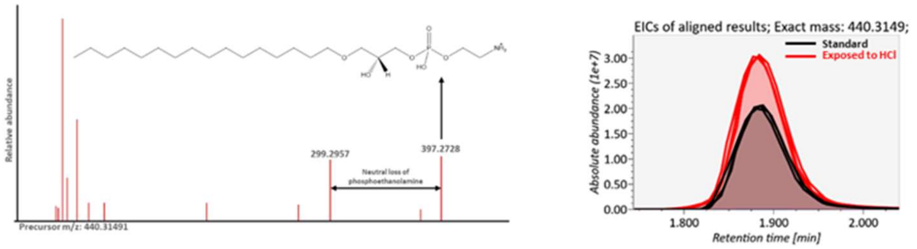

PE O-34:1 (PE O-16:0/18:1) (Retention time 6.572 min; *m/z* 704.55859; Adduct form [M+H]$^+$)

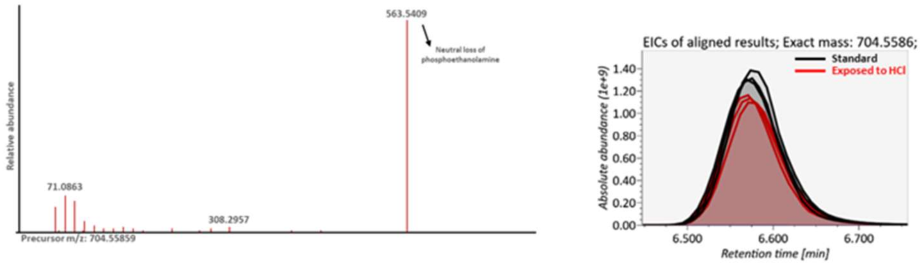

Supplementary Fig. 24. Annotation of plasmanyl phospholipids

MS/MS spectra and chromatogram of plasmanyl phospholipid standards used for lipid annotation. Corresponding chemical structures of fragments were indicated by arrows in MS/MS spectra. Plasmanyl forms were relatively stable in an acidic environment compared to plasmenyl forms. The peak of plasmanyl form was not affected by exposure to HCl fume (red lines in each chromatogram).

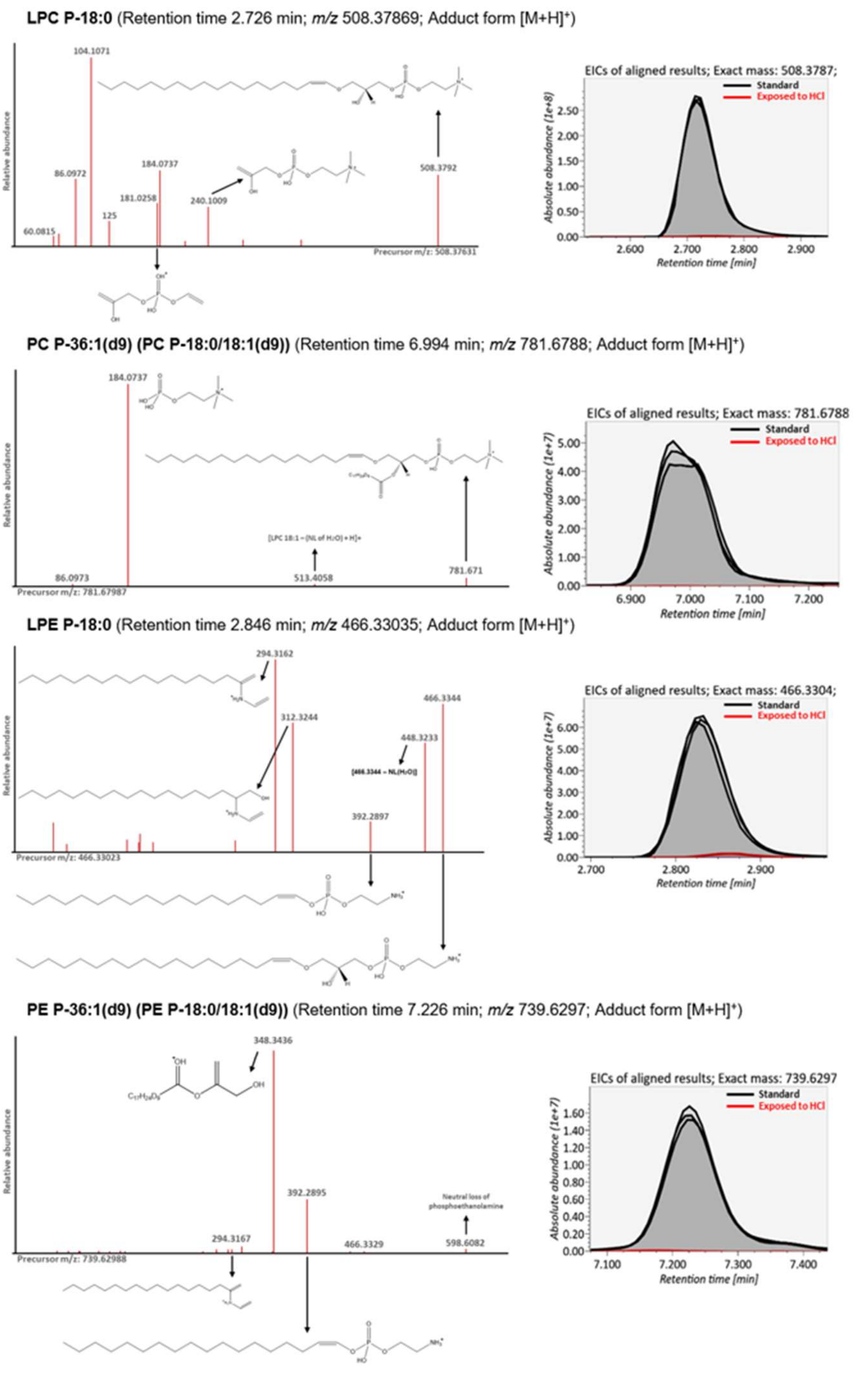

Supplementary Fig. 25. Annotation of plasmenyl phospholipids

MS/MS spectra and chromatogram of plasmenyl phospholipid standards used for lipid annotation. Corresponding chemical structures of fragments were indicated by arrows in MS/MS spectra. Plasmenyl forms were relatively unstable in an acidic environment compared to plasmanyl forms. The peak of plasmenyl form was disappeared after exposure to HCl fume (red lines in chromatogram).

Supplementary Tables

Supplementary Table 1. Population characteristics of human subcutaneous adipose tissue. Related to Fig. 8

Variable	
Age, *y*	38 ~ 82
Male, *n* (%)	44 (91.67%)
Female, *n* (%)	4 (8.33%)
BMI, kg/m^2	17.96 ~ 27.03
Height, *m*	1.47 ~ 1.78
Weight, *kg*	43.15 ~ 80

Supplementary Table 2. Primers used for qPCR and site-directed mutagenesis

Transcript	Forward primer	Reverse primer
Tmem86a	CCTTATCAAGTGCCTGCCCA	GGCAAACATCAGGAGACCATGT
Tmem86b	TGATCAAGTGCCAGCCCATT	AAGGCTGCCGTGCCATAAAA
Emr1	GTGACTCACCTTGTGGTCCT	CAGACACTCATCAACATCTGCG
Plin1	GAGTCAGCGACAGCTTCTTC	CTTGACGAGAAGCGACCTT
Pdgfra	AGCAGGCAGGGCTTCAACGG	ACACAGTCTGGCGTGCGTCC
Dio2	CCAGCACCGGAAAGAGGAAA	TCCTTGCACCATGACCCAAA
Cox8b	TGCGAAGTTCACAGTGGTTC	TCAGGGATGTGCAACTTCA
Ucp1	TGGCCTCTCAGTGGATGTG	CGTGGTCTCCCAGCATAGAAG
Pgc1a	ACACCTGTGACGCTTTCGCTG	AAGGACACGCTGTCCCATGA
Ppia	GTGGTCTTTGGGAAGGTGAA	TTACAGGACATTGCGAGCAG
Pde3b	GGATCGCAGCAGTGGTAAGA	AGCAGACACTGGTACATGGC
Tmem86a D82A	CTGCTGTGGGTGCTGCCTTCCTCATC	AAAAGACAAGTCCCACGAAGATGAGGG
Tmem86a D190A	TCATCCTCTCGGCCCTGACCATCGC	AGAGCAGAGCACCACCGCCGG

Supplementary Figure 14c

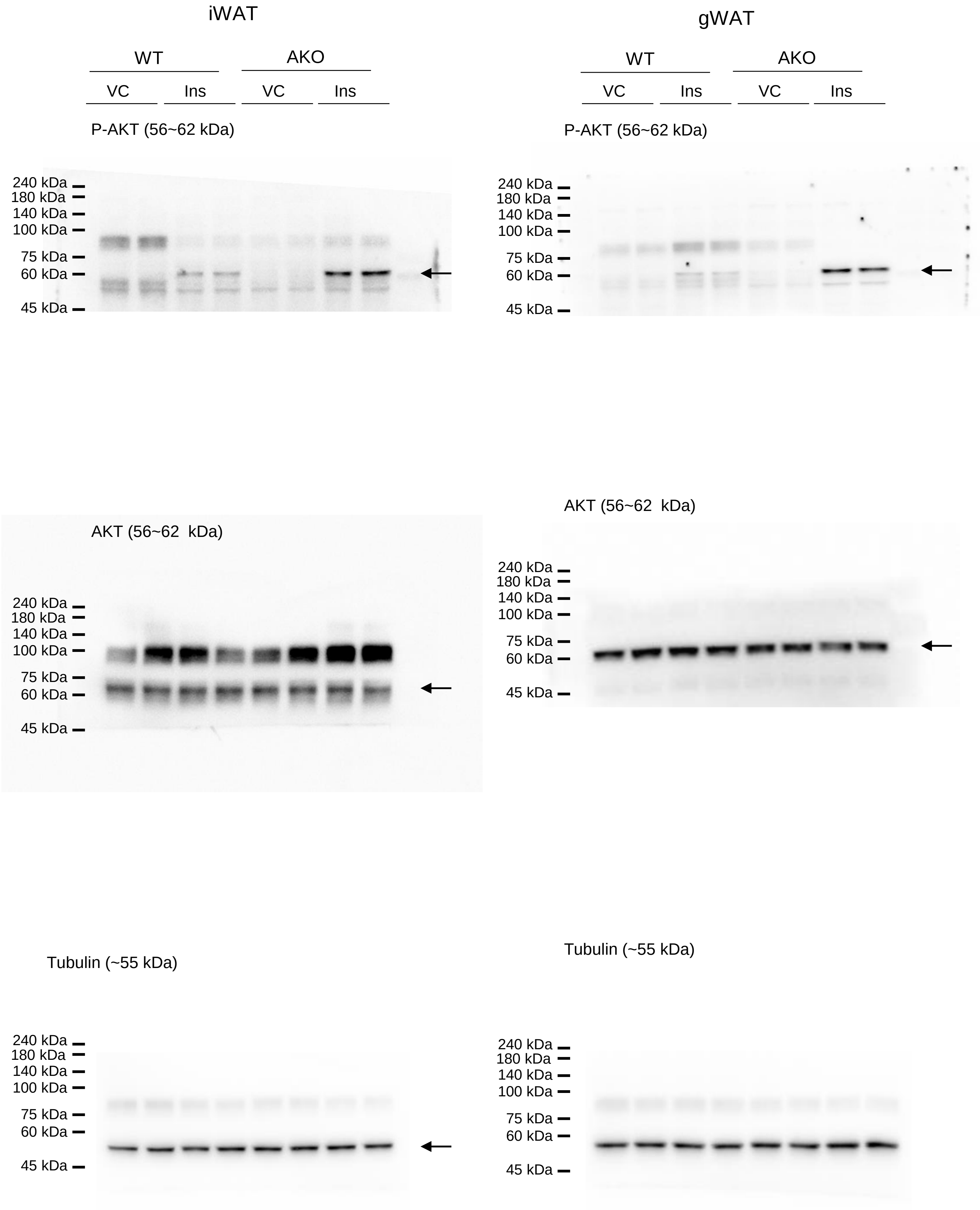

Supplementary Figure 18c

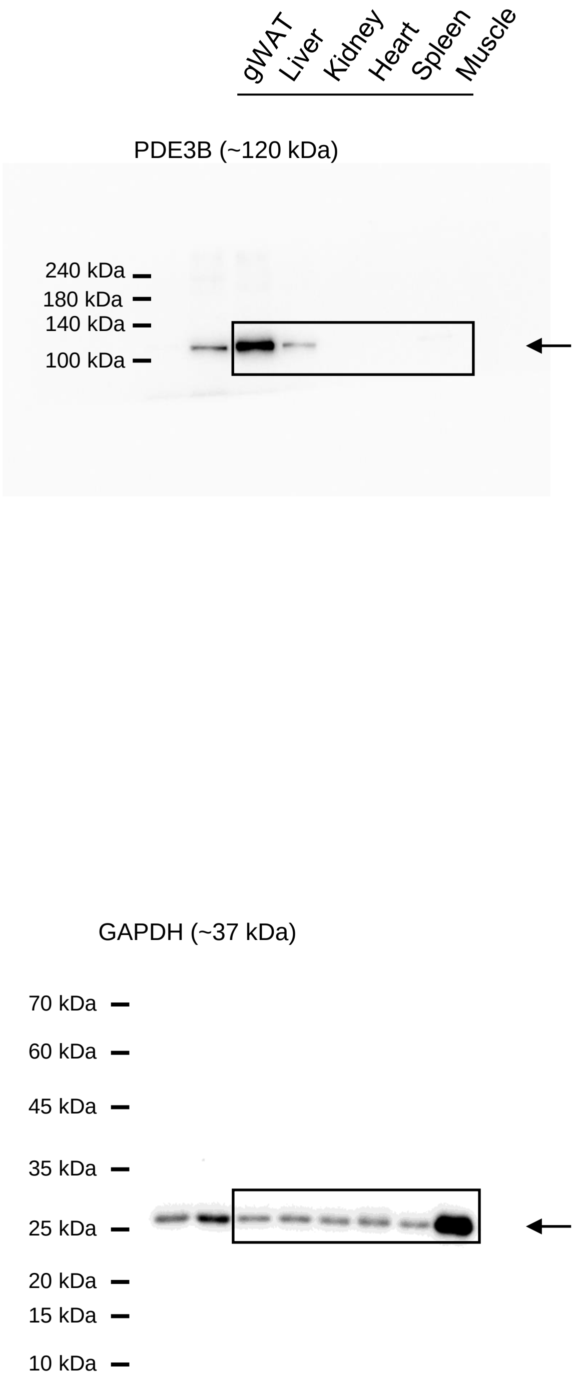

Supplementary Fig. 19a.

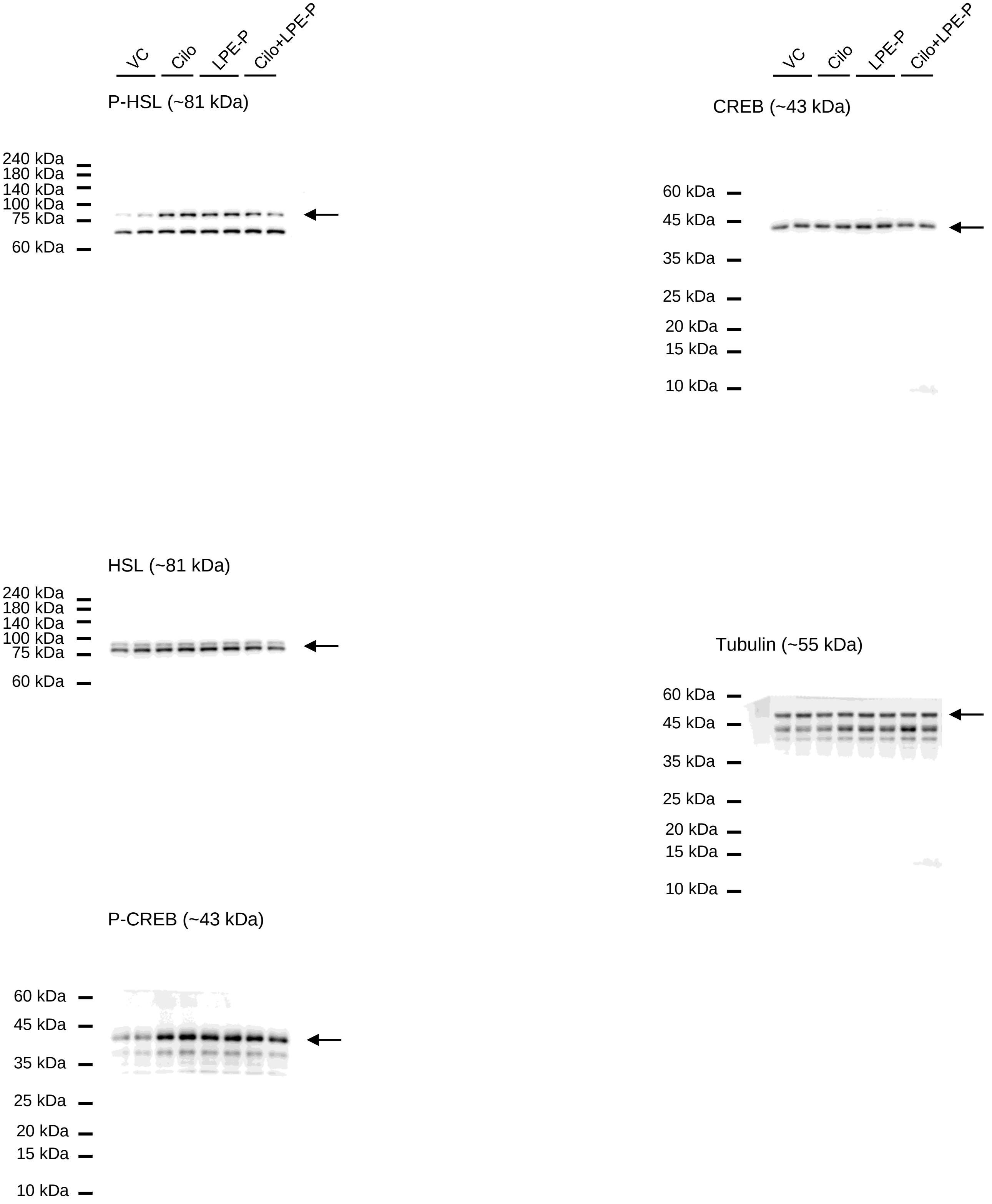

Supplementary Figure 19b

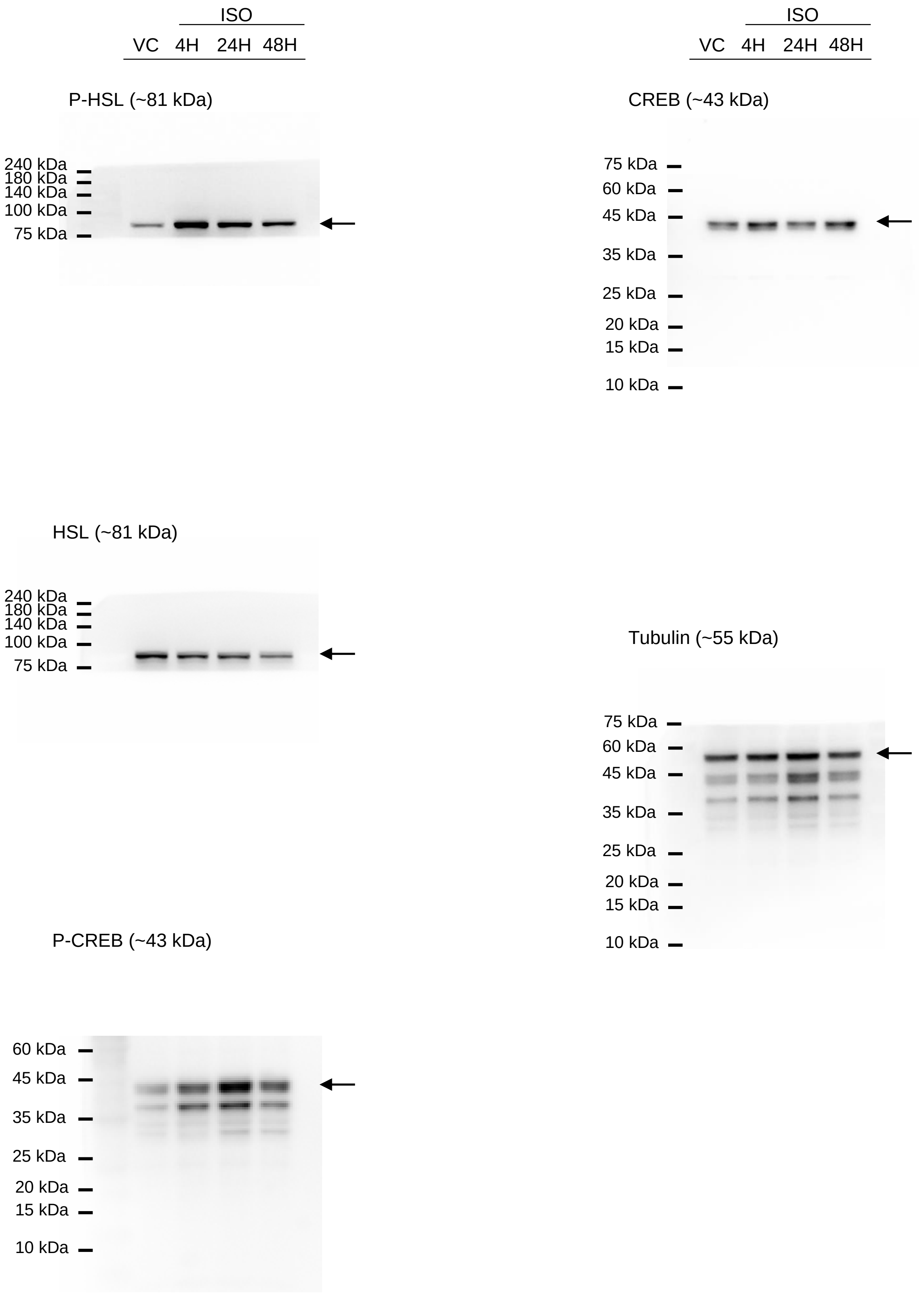

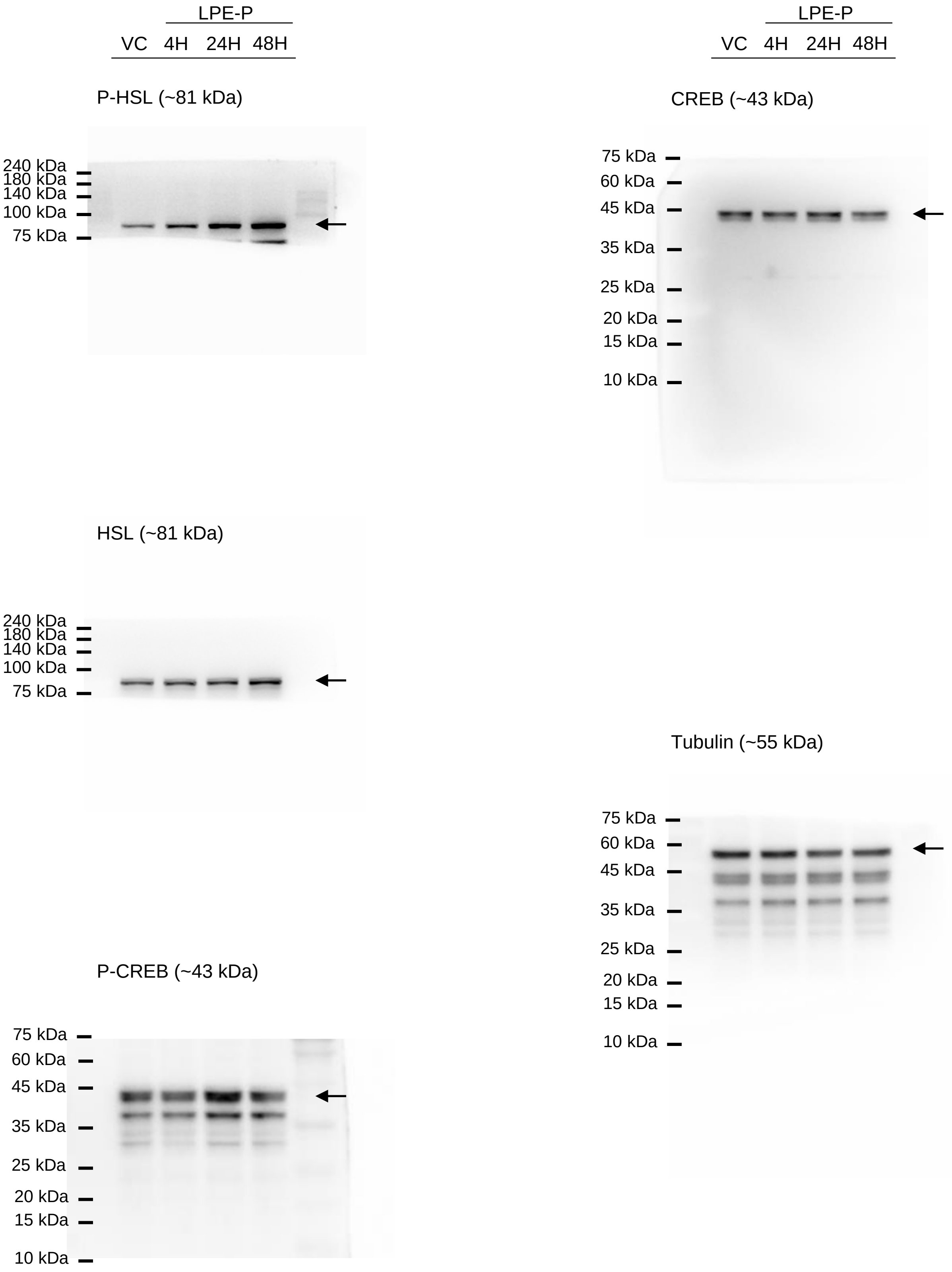

LPE-P
VC
4H
24H
48H
P-HSL (~81 kDa)
240 kDa
180 kDa
140 kDa
100 kDa
75 kDa
HSL (~81 kDa)
240 kDa
180 kDa
140 kDa
100 kDa
75 kDa
P-CREB (~43 kDa)
75 kDa
60 kDa
45 kDa
35 kDa
25 kDa
20 kDa
15 kDa
10 kDa
LPE-P
VC
4H
24H
48H
CREB (~43 kDa)
75 kDa
60 kDa
45 kDa
35 kDa
25 kDa
20 kDa
15 kDa
10 kDa
Tubulin (~55 kDa)
75 kDa
60 kDa
45 kDa
35 kDa
25 kDa
20 kDa
15 kDa
10 kDa

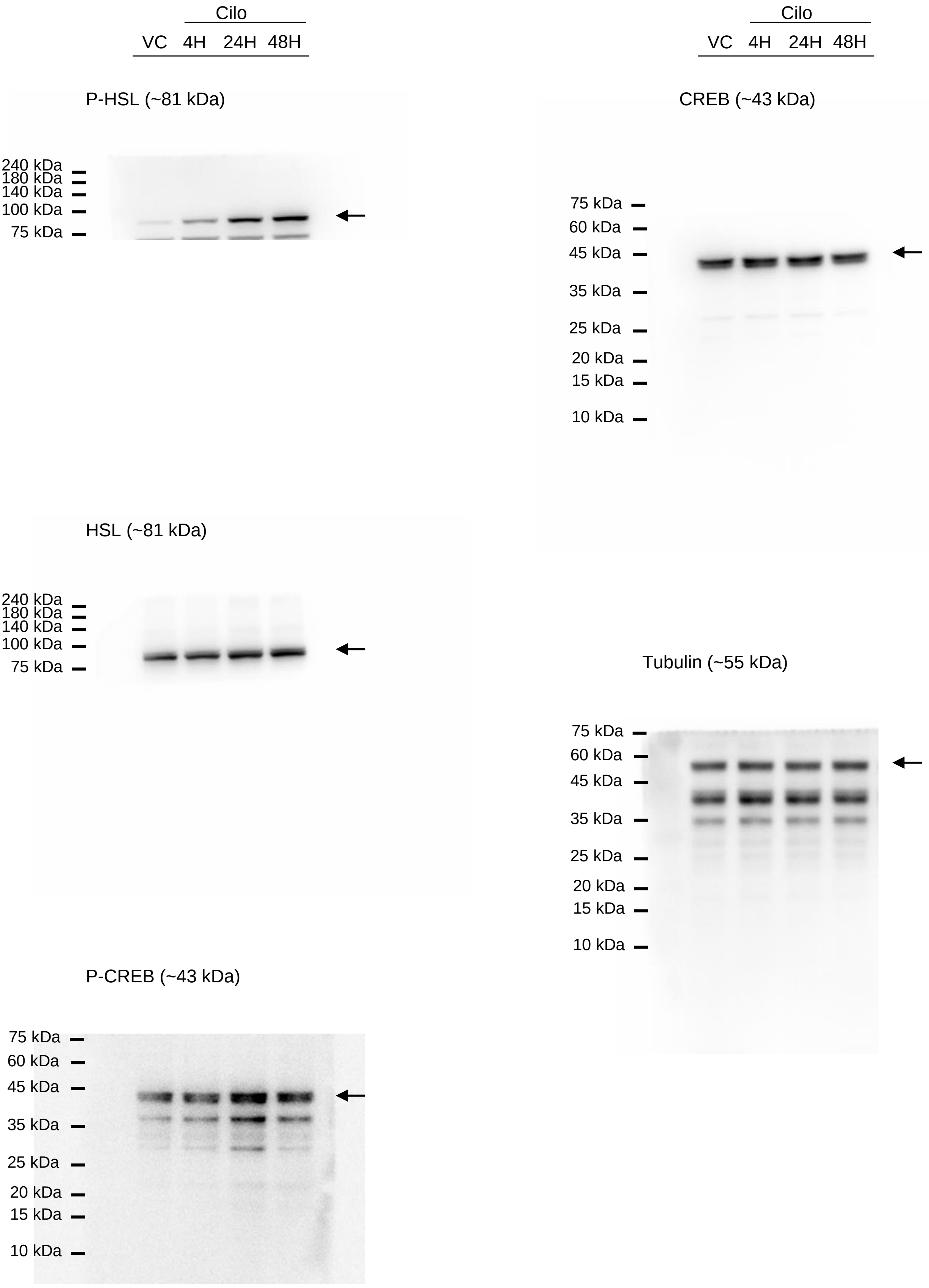
Cilo
VC 4H 24H 48H
P-HSL (~81 kDa)
240 kDa
180 kDa
140 kDa
100 kDa
75 kDa
HSL (~81 kDa)
240 kDa
180 kDa
140 kDa
100 kDa
75 kDa
P-CREB (~43 kDa)
75 kDa
60 kDa
45 kDa
35 kDa
25 kDa
20 kDa
15 kDa
10 kDa
Cilo
VC 4H 24H 48H
CREB (~43 kDa)
75 kDa
60 kDa
45 kDa
35 kDa
25 kDa
20 kDa
15 kDa
10 kDa
Tubulin (~55 kDa)
75 kDa
60 kDa
45 kDa
35 kDa
25 kDa
20 kDa
15 kDa
10 kDa
