# Supplementary material for: Adipocyte lysoplasmalogenase TMEM86A regulates plasmalogen homeostasis and protein kinase A-dependent energy metabolism
Source: Nat Commun. 2022 Jul 14;13:4084. doi: 10.1038/s41467-022-31805-3 (PMC9283435; doi:10.1038/s41467-022-31805-3)
Supplement: Supplementary file 3 — Description of Additional Supplementary Files [file 41467_2022_31805_MOESM3_ESM.pdf]

Description of Additional Supplementary Files

File Name: Supplementary Data 1
Description: **Detailed information for untargeted phospholipid profiling of C3H10T1/2 adipocytes overexpressing TMEM86A or mock-controls. Related to Fig.1**
Lipid species identified from untargeted phospholipid profiling of C3H10T1/2 adipocytes overexpressing TMEM86A or mock-controls along with experimental m/z, retention time, fold change, and statistical significance values. Statistical analysis was performed using normalized intensity. p-values and FDR values were determined using the unpaired, two-tailed t-test.

File Name: Supplementary Data 2
Description: **Detailed information for untargeted phospholipid profiling of adipose tissue from WT and TMEM86A AKO mice. Related to Fig. 3**
Lipid species identified from untargeted phospholipid profiling of adipose tissue from WT and TMEM86A AKO mice along with experimental m/z, retention time, fold change, and statistical significance values. Statistical analysis was performed using normalized intensity. p-values and FDR values were determined using the unpaired, two-tailed t-test.

File Name: Supplementary Data 3
Description: **Detailed information for untargeted phospholipid profiling of human subcutaneous adipose tissue. Related to Fig. 8**
Lipid species identified from the untargeted phospholipid profiling of human subcutaneous adipose tissue along with experimental m/z, retention time, and statistical significance. Statistical analysis was performed using normalized intensity. p-values and FDR values were determined using one-way ANOVA and Tukey's HSD post-hoc analysis.
