# Supplementary material for: Adipocyte lysoplasmalogenase TMEM86A regulates plasmalogen homeostasis and protein kinase A-dependent energy metabolism
Source: Nat Commun. 2022 Jul 14;13:4084. doi: 10.1038/s41467-022-31805-3 (PMC9283435; doi:10.1038/s41467-022-31805-3)
Supplement: Supplementary file 7 — Reporting Summary [file 41467_2022_31805_MOESM7_ESM.pdf]

nature portfolio

Corresponding author(s): Yun-Hee Lee

Last updated by author(s): Jun 9, 2022

Reporting Summary

Nature Portfolio wishes to improve the reproducibility of the work that we publish. This form provides structure for consistency and transparency in reporting. For further information on Nature Portfolio policies, see our Editorial Policies and the Editorial Policy Checklist.

Statistics

For all statistical analyses, confirm that the following items are present in the figure legend, table legend, main text, or Methods section.

n/a	Confirmed	
☐	☒	The exact sample size (*n*) for each experimental group/condition, given as a discrete number and unit of measurement
☐	☒	A statement on whether measurements were taken from distinct samples or whether the same sample was measured repeatedly
☐	☒	The statistical test(s) used AND whether they are one- or two-sided<br>*Only common tests should be described solely by name; describe more complex techniques in the Methods section.*
☒	☐	A description of all covariates tested
☒	☐	A description of any assumptions or corrections, such as tests of normality and adjustment for multiple comparisons
☐	☒	A full description of the statistical parameters including central tendency (e.g. means) or other basic estimates (e.g. regression coefficient) AND variation (e.g. standard deviation) or associated estimates of uncertainty (e.g. confidence intervals)
☐	☒	For null hypothesis testing, the test statistic (e.g. *F*, *t*, *r*) with confidence intervals, effect sizes, degrees of freedom and *P* value noted<br>*Give P values as exact values whenever suitable.*
☒	☐	For Bayesian analysis, information on the choice of priors and Markov chain Monte Carlo settings
☒	☐	For hierarchical and complex designs, identification of the appropriate level for tests and full reporting of outcomes
☒	☐	Estimates of effect sizes (e.g. Cohen's *d*, Pearson's *r*), indicating how they were calculated

Our web collection on statistics for biologists contains articles on many of the points above.

Software and code

Policy information about availability of computer code

Data collection	EvolutionCapt software ver 17.03 was used for capturing Immunoblot images.<br>Bio-Rad CFX Maestro 1.1 software ver 4.1.2433.1219 was used to acquire qPCR data.<br>Nikon Elements (NIS BR Analysis ver 5.10.00), Zeiss Zen software (ZEN 3.0 Blue edition) were used to acquire microscopy images.<br>BD FACSDiva 8.0 software was applied to acquire flow cytometry data.<br>SkanIt software ver 5.0 was used to measure the absorbance for BCA assay, cAMP ELISA assay, PDE3B activity assay, and TTC assay.<br>Thermofisher Scientific Xcalibur Software was used for acquisition of untargeted lipidomics LC-MS data.<br>Agilent MassHunter Workstation Data Acquisition ver B8023.0 was used for acquisition of LPE P-18:0 targeted LC-MS data.
Data analysis	GraphPad Prism7 software ver 7.00 was used for statistical test and visualizing graph.<br>NIH ImageJ software ver 1.52a was used to quantify immunoblot images.<br>FlowJo software ver 10.5.3 was applied to analyze flow cytometry data.<br>AssayFitPro ver 1.31 was used to obtain intracellular cAMP levels.<br>Agilent Wave software ver 2.6.0.31 was used to measure oxygen consumption rates.<br>AF-Q5BLD1-F1-model_v2 was used to create the 3D model of human TMEM86A protein.<br>Ggplot2 package (ver 3.3.3) was used to create volcano plots in R.<br>MS-DIAL ver 4.60 was applied to pre-process untargeted lipidomics LC-MS data.<br>MassHunter Quantitative Analysis ver B.07.00 was used for calculation of peak area of LPE P-18:0 in mouse serum.<br>El-MAVEN ver 0.12.0 was used for quantification of LPE P-18:0 in cell culture medium.<br>MetaboAnalyst 5.0 was used for statistical analysis of LC-MS data.

For manuscripts utilizing custom algorithms or software that are central to the research but not yet described in published literature, software must be made available to editors and reviewers. We strongly encourage code deposition in a community repository (e.g. GitHub). See the Nature Portfolio guidelines for submitting code & software for further information.

March 2021

Data

Policy information about availability of data

All manuscripts must include a data availability statement. This statement should provide the following information, where applicable:

- Accession codes, unique identifiers, or web links for publicly available datasets
- A description of any restrictions on data availability
- For clinical datasets or third party data, please ensure that the statement adheres to our policy

The RNA-seq data used in this study are available in Gene Expression Omnibus (GEO) database under accession number GSE182930. Global Transcriptome profiling conducted by Dahlman (PMID:28570579) were analyzed in the study and are publicly available in GEO database under accession number GSE94753. The Lipidomics data generated in this study have been deposited in a public database, MetaboLights under accession number MTBLS4703. MTBLS4703 is currently in the 'In Curation' status. ID and password are provided in the data availability statement to enable any access to MTBLS4703 prior to the release date (ID: yunyochl@snu.ac.kr, PW: NCOMMS-22-00143). The remaining data are available within the Article, Supplementary Information or Source Data files. Source data are provided with this paper.

Field-specific reporting

Please select the one below that is the best fit for your research. If you are not sure, read the appropriate sections before making your selection.

☒ Life sciences ☐ Behavioural & social sciences ☐ Ecological, evolutionary & environmental sciences

For a reference copy of the document with all sections, see nature.com/documents/nr-reporting-summary-flat.pdf

Life sciences study design

All studies must disclose on these points even when the disclosure is negative.

Sample size	Sample sizes were chosen to obtain statistical significance and reproducibility, based on previous experiments and experience, and common usage in the research field of in vivo metabolic phenotyping and cellular metabolism. In our previously published studies (Nature Metabolism, PMID:33758424), similar experiments were conducted with similar sample size (sample size, n =3-5 for cell-based assay, n= 6-14 for in vivo experiments).
Data exclusions	No data were excluded in any of the in vivo or in vitro experiments
Replication	All data are the result of independently-repeated experiments with independent biological sample. Experiments were repeated independently a minimum of three times with similar results in repeated experiments. All attempts at replication were successful assuming the technical reliability was acceptable within the experiment. Data reported in the manuscript are from biological replicates.
Randomization	All animals in our experiments were randomly allocated into different groups. In KO mouse study, randomization was applied to the mice carrying the same genotype. We randomly assigned experimental animals to treatment categories within the same genotype groups as genotype (KO vs WT) is a predetermined characteristic. For human samples, we randomly recruited patients who needed abdominal surgery and gave informed consent for sample use for research. There was no random selection process involved in this study for clinical samples, but the samples were grouped based on BMI.
Blinding	The investigators were blinded during the experiments and assessment of the outcome. Blinding was not performed during animal allocation to diet or treatment groups so that we could ensure each group had appropriate interventions.

Reporting for specific materials, systems and methods

We require information from authors about some types of materials, experimental systems and methods used in many studies. Here, indicate whether each material, system or method listed is relevant to your study. If you are not sure if a list item applies to your research, read the appropriate section before selecting a response.

Materials & experimental systems

n/a	Involved in the study	
☐	☒	Antibodies
☐	☒	Eukaryotic cell lines
☒	☐	Palaeontology and archaeology
☐	☒	Animals and other organisms
☐	☒	Human research participants
☒	☐	Clinical data
☒	☐	Dual use research of concern

Methods

n/a	Involved in the study	
☒	☐	ChIP-seq
☐	☒	Flow cytometry
☒	☐	MRI-based neuroimaging

Antibodies

Antibodies used

1. Mouse monoclonal B-actin Antibody, Santa Cruz, cat#sc-47778, RRID: AB_2714189, Lot#C1919; 1:1000 dilution; clone C4
2. Mouse monoclonal MCAD Antibody, Santa Cruz, cat#sc-365030, RRID: AB_10841423, Lot#L0211; 1:1000 dilution; clone A-4
3. Mouse monoclonal Phospho-AKT1/2/3 Antibody, Santa Cruz, cat#sc-514032, RRID:AB_2861344, Lot#L1520; 1:1000 dilution; C-11
4. Mouse monoclonal AKT1/2/3 Antibody, Santa Cruz, cat#sc-81434, RRID: AB_1118808, Lot#L1520; 1:1000 dilution; 5C10
5. Mouse monoclonal Total OXPHOS cocktail Antibody, Abcam, cat#ab110413, RRID: AB_2629281, Lot#P3338; 1:1000 dilution
6. Rabbit polyclonal HSL Antibody, Cell Signaling, cat#4107S, RRID:AB_2296900, Lot#3; 1:1000 dilution
7. Rabbit polyclonal Phospho-HSL Antibody, Cell Signaling, cat#45804S, RRID:AB_2893315, Lot#1; 1:1000 dilution
8. Rabbit polyclonal alpha/beta-tubulin Antibody, Cell Signaling, cat#2148S, RRID: AB_2288042, Lot#8; 1:1000 dilution
9. Rabbit monoclonal F4/80 Antibody, Cell Signaling, cat#30325S, RRID: AB_2798990, Lot#1; 1:1000 dilution; clone D4C8V
10. Rabbit monoclonal COX IV Antibody, Cell Signaling, cat#4850S, RRID: AB_2085424, Lot#10; 1:1000 dilution; clone 3E11
11. Rabbit monoclonal CREB Antibody, Cell Signaling, cat#9197S, RRID: AB_331277, Lot#17; 1:1000 dilution; clone 48H2
12. Rabbit monoclonal Phospho-CREB Antibody, Cell Signaling, cat#9198S, RRID: AB_2561044, Lot#18; 1:1000 dilution; clone 87G3
13. Rabbit polyclonal Phospho-(Ser/Thr) PKA Substrate Antibody, Cell Signaling, cat#9621S, RRID:AB_330304, Lot#15; 1:1000 dilution
14. Rat monoclonal PDE3B Antibody, Invitrogen, cat#14-1973-82, RRID: AB_1603296, Lot#2399929; 1:500 dilution; clone SMCP3B
15. Rabbit monoclonal PDI Antibody, Cell Signaling, cat#3501S, RRID: AB_2156433, Lot#3; 1:200 dilution; clone C81H6
16. Horseradish Peroxidase-conjugated Goat anti-rabbit IgG (H+L) Secondary Antibody, Invitrogen, cat#31460, RRID: AB_228341, 1:5000 dilution
17. Horseradish Peroxidase-conjugated Goat anti-mouse IgG, light chain specific Secondary Antibody, Jackson ImmunoResearch, cat#115-035-174, RRID: AB_2338512, 1:1000 dilution
18. APC/Cyanine7 anti-mouse CD45 Antibody, Biolegend, cat# 103116, RRID: AB_312981, Lot#B290026; 1:80 dilution; clone 30-F11
19. Brilliant Violet 711™ anti-mouse CD64 (FcγRI) Antibody, Biolegend, cat#139311, RRID: AB_2563846, Lot#B312770; 1:20 dilution; clone X54-5/7.1
20. FITC anti-mouse/human CD11b Antibody, Biolegend, cat#101206, RRID: AB_312789, Lot#B224362; 1:200 dilution; clone M1/70
21. APC anti-mouse CD11c Antibody, Biolegend, cat#117310, RRID: AB_313779, Lot#B331091, 1: 80 dilution; clone N418
22. Brilliant Violet 421™ anti-mouse CD206 (MMR) Antibody, Biolegend, cat#141717, RRID: AB_2562232, Lot#B342526, 1: 20 dilution; clone C068C2
23. Goat anti-Rabbit IgG (H+L) Cross-Adsorbed Secondary Antibody, Alexa Fluor™ 594, Invitrogen, cat#A-11012, RRID: AB_2534079; 1:500 dilution

Validation

The following antibodies have been validated by the manufacturer for Western Blotting and immunofluorescence or flow cytometry.

1. https://www.scbt.com/p/beta-actin-antibody-c4
Reactivity: mouse, rat, human, avian, bovine, canine, porcine, and rabbit
Application: Western Blotting, Immunoprecipitation, Immunofluorescence, Immunocytochemistry, Immunohistochemistry, and solid phase ELISA

2. https://www.scbt.com/p/mcad-antibody-a-4
Reactivity: mouse, rat and human
Application: Western Blotting, Immunoprecipitation, Immunofluorescence, and solid phase ELISA

3. https://www.scbt.com/p/p-akt1-2-3-antibody-c-11
Reactivity: mouse, rat and human
Application: Western Blotting, Immunoprecipitation, Immunofluorescence, Immunohistochemistry, and solid phase ELISA

4. https://www.scbt.com/p/akt1-2-3-antibody-5c10
Reactivity: mouse, rat, human and canine
Application: Western Blotting and Immunoprecipitation

5. https://www.abcam.com/total-oxphos-rodent-wb-antibody-cocktail-ab110413.html
Reactivity: mouse, rat, cow, human, and Cynomolgus monkey
Application: Western Blotting

6. https://www.cellsignal.com/products/primary-antibodies/hsl-antibody/4107
Reactivity: human and mouse
Application: Western Blotting, Immunoprecipitation, and Immunofluorescence

7. https://media.cellsignal.com/coa/45804/1/45804-lot-1-coa.pdf
Reactivity: human and mouse
Application: Western Blotting

8. https://media.cellsignal.com/coa/2148/8/2148-lot-8-coa.pdf
Reactivity: human, mouse, rat, monkey, zebrafish, and bovine
Application: Western Blotting, Immunohistochemistry, Immunofluorescence, and Flow Cytometry

9. https://media.cellsignal.com/coa/30325/1/30325-lot-1-coa.pdf
Reactivity: mouse
Application: Western Blotting, Immunoprecipitation, and Immunofluorescence

10. https://media.cellsignal.com/coa/4850/10/4850-lot-10-coa.pdf
Reactivity: Human, Rat, Monkey, Zebrafish, Bovine, and Pig
Application: Western Blotting, Immunoprecipitation, Immunofluorescence, Immunohistochemistry, and Flow Cytometry

Citation: PMID: 28878358, PMID:30258205, PMID: 33758424
Reactivity against mus musculus samples has been reported in the following studies cited above.

11. https://media.cellsignal.com/coa/9197/19/9197-lot-19-coa.pdf
Reactivity: Human, Mouse, Rat, Monkey, and D. melanogaster
Application: Western Blotting, Immunoprecipitation, Immunofluorescence, Immunohistochemistry, Flow Cytometry, and Chromatin IP

12. https://media.cellsignal.com/coa/9198/18/9198-lot-18-coa.pdf
Reactivity: Human, Mouse, and Rat
Application: Western Blotting, Immunoprecipitation, Immunofluorescence, Immunohistochemistry, Flow Cytometry, Chromatin IP, and Chromatin IP-seq

13. https://media.cellsignal.com/coa/9621/15/9621-lot-15-coa.pdf
Reactivity: All
Application: Western Blotting, Immunoprecipitation, Immunohistochemistry, and Peptide ELISA
Citation: PMID: 28637651, PMID: 27874008
Reactivity against mus musculus samples has been reported in the following studies cited above.

14. https://www.thermofisher.com/antibody/product/PDE3B-Antibody-clone-SMCP3B-Monoclonal/14-1973-82
Reactivity: Mouse
Application: Western Blotting

15. https://media.cellsignal.com/coa/3501/3/3501-lot-3-coa.pdf
Reactivity: Human, Mouse, Rat, Monkey
Application: Western Blotting, Immunofluorescence, and Immunohistochemistry

16. https://www.thermofisher.com/antibody/product/Goat-anti-Rabbit-IgG-H-L-Secondary-Antibody-Polyclonal/31460

17. https://www.jacksonimmuno.com/catalog/products/115-035-174

18. https://www.biolegend.com/en-us/products/apc-cyanine7-anti-mouse-cd45-antibody-2530
Reactivity: Mouse;
Application: Flow Cytometry

19. https://www.biolegend.com/en-us/products/brilliant-violet-711-anti-mouse-cd64-fcgammari-antibody-9920;
Reactivity: Mouse
Application: Flow Cytometry

20. https://www.biolegend.com/en-us/products/fitc-anti-mouse-human-cd11b-antibody-347
Reactivity: Mouse, Human
Application: Flow Cytometry

21. https://www.biolegend.com/en-us/products/apc-anti-mouse-cd11c-antibody-1813
Reactivity: Mouse
Application: Flow Cytometry

22. https://www.biolegend.com/en-us/products/brilliant-violet-421-anti-mouse-cd206-mmr-antibody-8638
Reactivity: Mouse
Application: Flow Cytometry and Immunohistochemistry

23. https://www.thermofisher.com/antibody/product/Goat-anti-Rabbit-IgG-H-L-Cross-Adsorbed-Secondary-Antibody-Polyclonal/A-11012

Eukaryotic cell lines

Policy information about cell lines

Cell line source(s)	HEK293T cells (human, ATCC CRL-3216)<br>C3H10T1/2 cells (mouse, ATCC CCL-226)
Authentication	HEK293T cells had been authenticated by the provider (ATCC) using STR profiling. We further authenticated the cell line by examining the morphological characteristics of the cell line by microscope. C3H10T1/2 were obtained from ATCC and the adipogenic identity of C3H10T1/2 cell line has been validated in the lab by its morphological characteristics and adipocyte marker expressions via RT-qPCR and western blotting.
Mycoplasma contamination	Cell lines were previously determined to be free from Mycoplasma contamination.<br>No indication of contamination was observed during experiments.
Commonly misidentified lines<br>(See ICLAC register)	No commonly misidentified cell lines were used.

Animals and other organisms

Policy information about studies involving animals; ARRIVE guidelines recommended for reporting animal research

Laboratory animals	Only adult male mice, aged 8-16 weeks were analyzed for this study. Mice were housed in a specific pathogen-free environment at 22 ± 1 °C, 50% ± 5 humidity, 12-h light/12-h dark cycle condition with free access to food and water. Tmem86a flox/flox mice were generated by crossing C57BL/6N-Tmem86atm1a(KOMP)Mbp mice (Korea Mouse Phenotyping Center, #MOP1812011) with ACTB-FLPe (B6.Cg-Tg(ACTFLPe)9205Dym/J: JAX stock #005703). Adipoq-Cre (B6.FVB-Tg(Adipoq-cre)1Evdr/J: stock#028020) mice were then bred with Tmem86a flox/flox mice to generate the following genotypes: Tmem86a flox/flox without adipoq-Cre (WT), Tmem86a flox/flox; adipoq-Cre (TMEM86A AKO). C57BL/6 mice (six weeks old, male) were purchased from Joongah Bio (Korea). For the diet-induced obesity model, mice at 8 weeks of age were fed with a high-fat diet (HFD) (60% fat, Research Diets) for eight weeks.
Wild animals	No wild animals were used in this study.
Field-collected samples	No field-collected samples were used in this study.
Ethics oversight	All of protocols related to animal experiment were approved by the Institutional Animal Care and Use Committees of Seoul National University (SNU-190821-2, SNU-201123-2).

Note that full information on the approval of the study protocol must also be provided in the manuscript.

Human research participants

Policy information about studies involving human research participants

Population characteristics	The specimens from patients undergoing abdominal surgery were collected randomly during archiving period. 48 participants were aged between 38 and 82 including both sexes. The height (m) and weight (kg) were ranged from 1.47 ~ 1.78 and 43.15 ~ 80, respectively. BMI (kg/m2) was ranged from 17.96 to 27.03. Samples were divided into 4 groups depending on BMI. Other information such as treatment category was not collected.
Recruitment	Patients diagnosed with prostate cancer (n = 32), kidney tumor (n = 14), or renal pelvis tumor (n = 2) agreed to donate their removed fat tissues for research purposes only and then intra-abdominal and subcutaneous fat tissues of recruited patients were collected during the surgery at Surgery Unit of Pusan National University Hospital. No self-selection bias is predicted, as BMI was the only parameter considered for recruitment.
Ethics oversight	This study was approved by the Institutional Review Board of Pusan National University Hospital (permit number: PNUH IRB 2101-019-099)

Note that full information on the approval of the study protocol must also be provided in the manuscript.

Flow Cytometry

Plots

Confirm that:

☒ The axis labels state the marker and fluorochrome used (e.g. CD4-FITC).

☒ The axis scales are clearly visible. Include numbers along axes only for bottom left plot of group (a 'group' is an analysis of identical markers).

☒ All plots are contour plots with outliers or pseudocolor plots.

☒ A numerical value for number of cells or percentage (with statistics) is provided.

Methodology

Sample preparation	Gonadal white adipose tissues were dissected, minced, and digested with collagenase type I in KRBB buffer containing 3% BSA at 37 °C. Red blood cells were lysed with RBC lysis buffer and SVF were then washed with KRBB buffer containing 1% BSA. SVF was stained with fluorescence labeled primary antibodies or control IgG for 20 min at room temperature.
Instrument	LSRFortessa X-20 Flow Cytometer was used for flow cytometry
Software	BD FACSDiva software was applied to acquire flow cytometry data. Flowjo software version 10.5.3 was used to analyze flow cytometry data.
Cell population abundance	All cells were first gated by FSC-A vs. SSC-A to exclude cell debris and then gated as singlets (FSC-A vs. FSC-H and SSC-A vs. SSC-H). Abundance of single cells were > 55% from the total recorded events. We analyzed about 100,000 live single cells per sample in this study. We then defined leukocytes by their expression of CD45. From leukocytes we identified total macrophages by their expression of CD11b and CD64. M1 macrophages were identified as CD11b+CD64+CD11c+ and M2 macrophages were identified as CD11b+CD64+CD206+. We did not perform sorting by fluorescence-activated cell sorting for our biological samples, but we performed analytic flow cytometry to collect forward-scatter, side scatter, and fluorescence signal data of the stained cells. We determined marker expressing cells computationally by comparing fluorescence signal data of samples to single surface marker-stained controls and unstained controls.

Populations were identified as described in the Methods and shown in Supplementary Fig. 15b.

Gating strategy

The gating strategy is provided in Supplementary Fig. 15b.
FSC-A vs. SSC-A gating was used to identify the distinct cells and to exclude debris.
Singlets were identified by FSC-A vs. FSC-H and SSC-A vs. SSC-H gating.

☒ Tick this box to confirm that a figure exemplifying the gating strategy is provided in the Supplementary Information.
